# Dengue virus causes changes of MicroRNA-genes regulatory network revealing potential targets for antiviral drugs

**DOI:** 10.1186/s12918-017-0518-x

**Published:** 2018-01-04

**Authors:** Mohamed Shahen, Zihu Guo, Akhtar Hussain Shar, Reham Ebaid, Qin Tao, Wenjuan Zhang, Ziyin Wu, Yaofei Bai, Yingxue Fu, Chunli Zheng, He Wang, Piar Ali Shar, Jianling Liu, Zhenzhong Wang, Wei Xiao, Yonghua Wan

**Affiliations:** 1 College of Life Science, Northwest A & F University, Yangling, Shaanxi 712100 China; 2 Center of Bioinformatics, Northwest A & F University, Yangling, Shaanxi 712100 China; 3 Zoology Department, Faculty of Science, Tanta University, Tanta, 31527 Egypt; 4 School of Environment and Safety Engineering, Jiangsu University, Jiangsu, 212013 China; 5 College of Life Science, Northwest University, Xi’an, Shaanxi 710069 China; 6 State Key Laboratory of New-tech for Chinese Medicine Pharmaceutical Process, Lianyungang, Jiangsu 222001 China

**Keywords:** Dengue virus, Immunity, miRNAs expression, Microarray, Mosquito

## Abstract

**Background:**

Dengue virus (DENV) is an increasing global health threat and associated with induction of both a long-lived protective immune response and immune-suppression. So far, the potency of treatment of DENV via antiviral drugs is still under investigation. Recently, increasing evidences suggest the potential role of microRNAs (miRNAs) in regulating DENV. The present study focused on the function of miRNAs in innate insusceptible reactions and organization of various types of immune cells and inflammatory responses for DENV. Three drugs were tested including antiviral herbal medicine ReDuNing (RDN), Loratadine (LRD) and Acetaminophen.

**Results:**

By the microarray expression of miRNAs in 165 Patients. Results showed that 89 active miRNAs interacted with 499 potential target genes, during antiviral treatment throughout the critical stage of DENV. Interestingly, reduction of the illness threats using RDN combined with LRD treatment showed better results than Acetaminophen alone. The inhibitions of DENV was confirmed by decrease concentrations of cytokines and interleukin parameters; like TNF-α, IFN-γ, TGF-β1, IL-4, IL-6, IL-12, and IL-17; after treatment and some coagulants factors increased.

**Conclusions:**

This study showed a preliminary support to suggest that the herbal medicine RDN combined with LRD can reduce both susceptibility and the severity of DENV.

**Electronic supplementary material:**

The online version of this article (10.1186/s12918-017-0518-x) contains supplementary material, which is available to authorized users.

## Background

Dengue fever disease (DF)is a mosquito-borne disease caused by Dengue virus (DENV) which has prominence in the past 20 years with an expanded geographic distribution of both the viruses and the mosquitoes [1]. Its spread rate was estimated as 390 million infections annually worldwide, and it is most prevalent in the developing countries [2]. DF is one of the mosquito-borne diseases which causes death all round equatorial and sub equatorial countries. The propagation of several life-threatening pathogens in human is caused by DF disease [3]. DF is caused by Dengue virus (DENV), a mosquito-borne flavivirus. DENV is a single positive-strand RNA virus of the family Flaviviridae, genus Flavivirus. Its innate immune system towards a variety of diseases in humans [4].

The symptoms of DF patients suffering dengue infection commonly exist during three to 7 days of hard flu-like sickness inclusive rise fever, nausea, puke, headache and body soreness [5]. After that viral infection innate and adaptive immune response are essential for the survival of the host infected by the DENV, the virus infection induce the immune system to generate type I IFN and proinflammatory cytokines parameters [6]. The host cell starts to defend against viral invasion through an appropriate innate immune response. Response to viral infection must be tightly regulated to defend rapidly against it, while minimizing inflammatory damage, that leads to cellular resistance to viral infection [7].

Several clinical trials were conducted to treat DENV and reduce the impact and symptoms, but until now no any licensed medicine could deal with DF. However, supportive care with a sedative, the replacement of fluids and bed rest can be useful in some cases [5, 8] recommended that a dosage of Acetaminophen 1 g every 8 h for 3 days dealing with DENV might be useful, while Loratadine (LRD) tablets is an orally effective medicine (ethyl4-(8-chloro-5,6-dihydro-11H-benzo, cyclohepta [1,2-b] pyridin-11-ylidene)-1-iperidinecarboxylate) [9], that could be used to treat antihistamine and relieve other allergy symptoms [10]. Additionnaly, ReDuNing (RDN) medicine a patented traditional antipyretic-detoxicate Chinese injection medicine, has been widely used as an anti-inflammatory and anti-infectious drug in Chinese clinics [11]. Dealing with the severity of dengue demands taking caution toward bodily fluids exchanges, while using proactive therapy for bleeding [12]. RDN medicine is composed of three herbs, *Artemisiae annuae* L. (genus Artemisia, Asteraceae), *Gardenia jasminoides* J.Ellis (genus Gardenia, Rubiaceae) and *Lonicera japonica* Thunb. (genus Lonicera, Caprifoliaceae) [13]. RDN injection medicine exhibits promising effects against enterovirus 71 in Vero monkey cells [14]. From our previous study, some methods were experimentally established to combat inflammation and provide a systems-based strategy to find the therapeutic mechanism of traditional Chinese medicine RDN dealing with the disease [13].

Nowadays miRNAs (miRNAs) has carried out numerous cautions and applications because several experimental studies have concluded that miRNAs could perform important roles in various biological processes including viral infection, differentiation, apoptosis, metabolism, cell proliferation, development, aging, signal transduction, as well as the development and progression of the human complex illness [15, 16]. Furthermore, miRNAs have close associations with the development, progression, and prognosis of many human illnesses [16, 17], furthermore, miRNAs are widely identified to be correlated with physiological processes: including apoptosis, differentiation, growth and stress responses, also pathological processes: including prognosis, initiation and progression of various illnesses [18]. miRNAs are small (18–25 base long) noncoding RNAs, which were identified as vital ingredients of immune precision and other biological processes, such as development infection and inflammation [19]. Indeed miRNAs have a primary function in the early differentiation of B cells and maintaining the regulatory T-cell lineage, additionally, they regulate the differentiation of dendritic cells and macrophages via toll-like receptors [20]. Recently, microRNAs have caused great excitement as diagnostic and therapeutic signatures of the DF [21]. However, arguments for the possibility of a major function of microRNAs in controlling target genes of DF were increased [22]. So, our studies on joint activation and inhibition of endogenous miRNAs try to fight against dengue fever that includes effects on the initial Immune reaction.

The present study aimed to evaluate the treatment activity of Acetaminophen, RDN and LRD against DF. In addition, the way of treatment using single or mixed therapy was tested.

## Methods

### Study enrollment and investigations

This study was performed a randomized and investigator blinded. We evaluated outpatients referred to Centre for Tropical Diseases, Guangzhou, China during March–December 2015. It was approved by the ethical committee at Guangzhou University hospital of Traditional Chinese Medicine (Guangzhou, China). Patients diagnosed as DF were recruited from first affiliated at hospital. Patients were divided into two phases; primary phase was indicator chosen through a small group of plasma specimen of 20 patients treated with Acetaminophen or RDN injection contaminated LRD tablet and 15 healthy volunteers. The secondary phase was an open evaluation with a big group number of plasma specimen from 165 patients [average (SD) age, 38 (14.11) years] and 45 healthy persons as a control [age, 39.19 (10.16) years].

### Diagnostic procedures

Tested DENV specific antibodies IgG and IgM in plasma samples were obtained from screened patients by ELISA antigen enzyme-linked immunosorbent assay (ELISA, Focus Technologies, Cypress, Ca, USA) [20]. Positive IgM cases were serologically confirmed by ELISA. Moreover, DV antigen and viral RNA were detected by a dot blot immunoassay and a dengue serotype specific reverse transcription PCR [23].

### Microarray

Three days post Acetaminophen or RDN contaminated LRD or non-treated infection patients group. Plasma samples were drawn in PAXgene tubes to maintain miRNAs integrity and provide a snapshot of in vivo blood expression while minimizing time and manipulation variations [24]. Total RNA was extracted by mirVana miRNAs isolation kit (Ambion, Austin, TX, USA), and was amplified and assorted according to the Affymetrix GeneChip Total Transcript Sense Target Labeling protocol [25]. Replicate miRNAs in duplicate samples were averaged and miRNAs with intensities of ≥50 in all samples from each group (DF-infected or treated groups) were selected for calculation of the normalization factor. Expressed data were median-normalized. Following normalization, significantly differentially expressed miRNAs were identified through Volcano Plot filtering as described by Cui and Churchill [26]. The detailed information of these differentially expressed miRNAs and correlation coefficient matrix for this data are listed in the Additional file 1 and the detailed result of volcano Plots filtering differentially expressed miRNAs are listed in the Additional file 2.

### Drugs and patients treatment

ReDuNing (RDN, Batch No. 100906) produced by Jiangsu Kanion Pharmaceutical Co., Ltd., (Jiangsu, China); Loratadine (LRD, batch LRD/0909180) produced by Vasudna Pharma. Chem. Ltd., China were used in the research for their potential activities against dengue virus inhibition. Experimental patients were randomly divided into 3 groups; model control (without treatment) received saline only, positive control (treated with 1.3 g Acetaminophen tablets) administered orally three times daily for 3 days, and third group treated with 10 mg LRD tablets and 20 ml RDN injection. RDN dose conversion was according to its clinical dose. LRD and RDN were administered to patients the day after stress orally and by intravenous injection, respectively, for 3 days.

### Network construction and analysis

To recognize the reaction mechanism of Acetaminophen and LRD with RDN as an herbal drug in the treatment of dengue fever, all active expression miRNAs and their targets were utilized to create interactions of miRNA and a predict gene were linked to each other if the gene was a predicted target of the miRNAs distributed in human. The potential miRNA were connected with those related target which were obtained from the PharmGKB database [27], and Therapeutic Targets Database (http://bidd.nus.edu.sg/group/bidd.htm).

In pharmaceutical systems, network consists of nodes and edges (connections between nodes), that is a mathematical, computable and quantifiable description of multiple relationships beneath the complicated biological systems [28]. To explore comprehensively the interrelationship between potential targets and miRNAs expressions which will help us to select out those specific targets genes correlated with DENV disease. In the resultant networks, miRNAs predicted targets genes and enrichment functions diseases were illustrated via nodes; while edges indicated the interactions between them.

Mi–T–P network was set up by overlaying the miRNAs-target gene network with related function pathway. Initially functional pathways defined by the KEGG database (Kyoto Encyclopedia of Genes and Genomes) (http://www.genome.jp/kegg/) [29] and then constructed by combining the predict targets and their related function pathways, the Database for Annotation, Visualization and Integrated Discovery (DAVID knowledgebase) (https://david.ncifcrf.gov/) [30] were used [30] to test for enriched DEmiRs targets function and to identify biological themes among them. For each miRNAs expressions, the DAVID web service provided a ranked list of functionally relevant annotation clusters that represent a summary of several annotation categories, including GO [30], KEGG [29] and BioCarta pathways [31]. In these networks, degree is used to characterize the connectedness of a node. The degree of a node is the number of edges associated with it. The topological properties of these networks were analyzed using the Network Analysis plugin and CentiScaPe 1.2 of Cytoscape 3.2.0, a popular bioinformatics package for biological network visualization and data integration, was used to generate all displayed networks [32].

To detect functional pathways or gene ontologies that are processing of differentially expressed miRNAs and putative targets of differentially expressed miRNAs after Dengue disease treated with Acetaminophen, RDN and LRD [19].

### Effect of antiviral inhibition on inflammatory immune response

The supernatants samples were collected from patients before treatment and 3 days post treatment with Acetaminophen, LRD and RDN. Concentrations of TNF-α, IFN-γ, TGF-β1, IL-4, IL-6, IL-12, and IL-17 in culture supernatants were measured by ELISA according to manufacturer’s instructions [33].

### Coagulations factor influences

Quantitative assessment of coagulations factor was performed in the plasma with DENV isolates using the ELISA Enzygnost (Dade Behring, Deerfield, IL, USA) according to the manufacturer’s instructions. Examination, we used plasma from untreated patients. In the case of the negative control, plasma was utilized. In parallel restrained survey in presence of hirudin (special thrombin restrained) was completed.

### Statistical analysis

The analyses of miRNA expression data for microarray and miRNA-Target gene networks were described in each section. Other statistical analysis of Coagulations factor influences and inflammatory immune response data were performed using Student’s t-test and data are expressed as means ± SE, and differences with probability level *P* ≤ 0.05 were considered statistically significant.

## Results

The herbal medicine is widely used in clinical practices for thousands of years with efficacy and safety. However, there were few studies on anti-dengue with herbs. In this study, we compare single therapy of Acetaminophen with a new regimen of intermittent sequential therapy with Acetaminophen, LRD and RDN in the treatment of dengue virus disease. This randomized clinical trial was performed from March to December 2015. Patients diagnosed clinically with DF were randomized in three groups: patients in arm, not received any treatment, in single therapy arm patients received Acetaminophen 1.3 g tablet three times daily and in other arm, patients received sequential regimen of LRD 10 mg tablet each day and RDN 20 ml injection once daily. We carried out the detailed analysis for (RDN single treatment, RDN with Acetaminophen and LRD with Acetaminophen) however, the results were not satisfactory (data not shown).

### Human miRNAs expression network and predicted target gene response to dengue virus infection

**Fig. 1:**
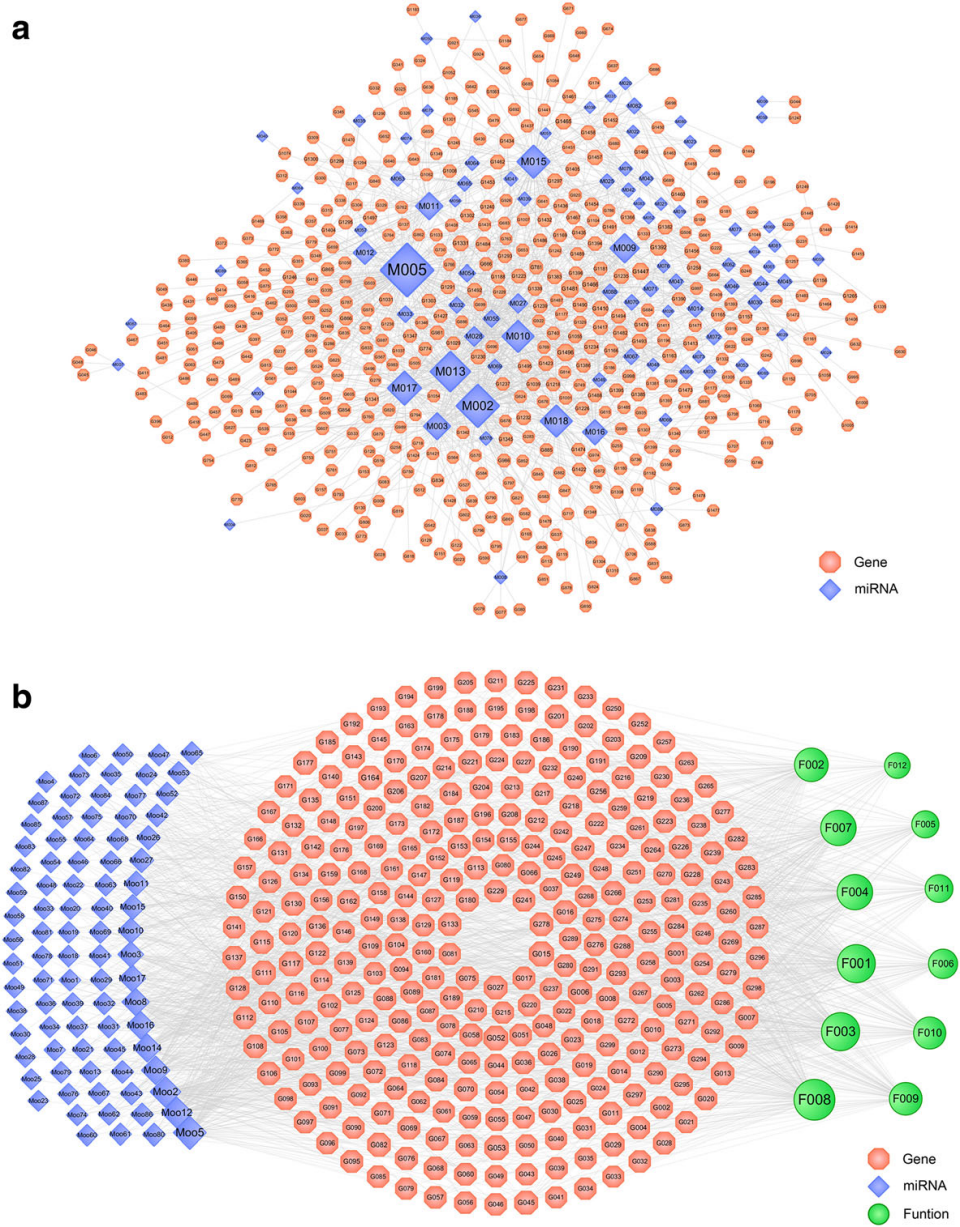
Network diagram of miRNAs expression and predicted target genes response to dengue virus infection without treatment (**a**) miRNAs-gene target relationship. A miRNA and a target are linked if the target protein is hit by the corresponding miRNA. **b** miRNAs-target-function pathway network. A miRNA and a target are linked if the target protein is hit by the corresponding miRNAs. Similarly, a target and a functional module are linked if the target is involved in this biological process. Node size is proportional to its degree and the letters are node labels

Figure 1a shows the miRNAs and their targets. In total, this network consists of 588 nodes (89 miRNAs and 499 targets) and 1497 edges. This indicates that many targets are hit by only one miRNA, while some targets can be adjusted by numerous ligands. In the interactions between miRNAs and targets, for those active miRNAs expression, M005 exhibited the highest number of target candidate (degree = 134), followed by M002 (degree = 102), M013 (degree = 93), M017 (degree = 73) and so on. As for the candidate targets, DDX3X (DEAD-Box Helicase 3, X-Linked) showed the highest degree (Degree = 18), with PTEN (Phosphatase and Tensin Homolog, Degree = 17), RORA (RAR Related Orphan Receptor A, Degree = 15), PPP3R1 (Protein Phosphatase 3 Regulatory Subunit B, Alpha, Degree = 14), suggesting that DDX3X and PTEN might be potential targets for dengue disease and they were modulated through related miRNAs. The detailed information of these miRNAs expressions and target genes response to DENV are listed in the Additional file 3: Table S1.

DDX3X plays an important role in the regulation of the cell proliferation, which indicated that it is involved in antiviral response against DENV infection [34], moreover DDX3X is able to stimulate the IFN promoter branches in DENV-infected cells and can be used as human immunodeficiency virus to control dengue infection. It has been suggested that the up-regulation of DDX3X may lead to treat dengue infection [35]. The suppression of PTEN gene represents a powerful strategy for ESCC therapy while PTEN has potent antiviral activity against both HIV-1 and dengue virus [36]. RORA has been implicated in several different cellular processes, including cell growth, translational control and nuclear–cytoplasmic shuttling [36]. It was previously proved that RORA played a critical role in the cellular response, such as inhibitor of NF-κB signaling in cell to achieve inhibitory effects on immune response programs [37, 38] Reported that RORA is a candidate transcription factor for suppressing the inflammatory response employed by H1N1 of human monocytes. PPP3R1 was reported to participate in several biological processes such as improve therapeutic strategy to treat autoimmune disorders and neurological disorders such as Alzheimer disease either responses of skeletal muscle and cardiac muscle [39].

Microarray analysis demonstrated that aberrant miRNAs expression was a remarkable characteristic in plasma after infection with DENV. Figure 1b showed that Mi-T-P network consisted of 87 plasma miRNAs which were differentially down-regulated, 399 candidate genes were up-regulated and their corresponding 12 functional annotations. M005 (degree = 71) exhibited the highest active miRNAs expression, followed by M012 (degree = 59), M002 (degree = 58) and M009 (degree = 45), indicating the multi-target properties of ingredients, which is the summary of the action mode of miRNAs expression. The targets with highest degree are listed in (Table 1). Among these targets, PTEN (Phosphatase and Tensin Homolog, degree = 21) showed the highest degree, that is PTEN could interact with the most nodes in the network, which plays a key role in regulating the network as the hub target. Moreover, PTEN was followed by DDX3X (DEAD-Box Helicase 3, X-Linked, degree = 19), ADRB2 (Adrenoceptor Beta 2, degree = 17) and VEGFA (Vascular Endothelial Growth Factor A, degree = 16) which are potential therapeutic targets genes for DENV. The detailed information of these miRNAs expressions, target genes and functional pathway are listed in the Additional file 4: Table S2. In summary, the inhibition of these six of 399 proteins (DDX3X, PTEN, RORA, PPP3R1, VEGFA, and ADRB2) may significantly decrease the replication of a DENV replicon.

**Table 1:** The highest target genes response to dengue virus infection

Gene symbol	Protein name	ID	Degree
PTEN	Phosphatase and Tensin Homolog	G015	21
DDX3X	DEAD-Box Helicase 3, X-Linked	G052	19
ADRB2	Adrenoceptor Beta 2	G053	17
VEGFA	Vascular Endothelial Growth Factor A	G006	16
PPP3R1	Protein Phosphatase 3 Regulatory Subunit B, Alpha	G117	16
MAP3K1	Mitogen-Activated Protein Kinase Kinase Kinase 1	G164	15
IGF1R	Insulin Like Growth Factor 1 Receptor	G038	14
RELA	RELA Proto-Oncogene, NF-KB Subunit	G045	14
NRAS	Neuroblastoma RAS Viral Oncogene Homolog	G209	14
CHUK	Conserved Helix-Loop-Helix Ubiquitous Kinase	G266	14
MAPK1	Mitogen-Activated Protein Kinase 1	G016	13
BCL10	B-Cell CLL/Lymphoma 10	G027	13
ADAM10	ADAM Metallopeptidase Domain 10	G111	13
RICTOR	RPTOR Independent Companion Of MTOR Complex 2	G130	13
MAPK9	Mitogen-Activated Protein Kinase 9	G177	13
CCDC88A	Coiled-Coil Domain Containing 88A	G019	12
PRKCE	Protein Kinase C Epsilon	G070	12

In addition, there are a number of targets involve in several DENV-associated biological processes and succeed functional annotation clustering analysis were reported (Table 2). These biological processes can be summarized into several functional modules; such as regulation of cell proliferation (which is belongs to the inflammatory regulation module), regulation of programmed cell death and apoptosis.

**Table 2:** Gene Ontology (GO) analysis of therapy target genes

Function Name	ID	Degree
Protein kinase	F008	118
regulation of cell proliferation	F003	102
regulation of programmed cell death and apoptosis	F001	100
response to cytokine stimulus	F004	86
protein amino acid phosphorylation	F007	86
immune response	F002	76
protein kinase cascade	F009	70
T cell receptor signaling pathway	F010	67
regulation of lymphocyte activation	F006	49
Toll-like receptor signaling pathway	F011	42
myeloid cell differentiation	F005	38
RIG-I-like receptor signaling pathway	F012	29

The degree shows scores of significantly enriched ‘Biological Process’ categories in GO relative to the target genes, (*p* value ≤ 0.05)

### Network construction and analysis for clinical treatment

In general, RDN Injection combined with LRD oral administration can induce the expression of miR-128a, miR-389 and miR-9 to suppress dengue virus replication. [40] Previously reported that in vivo miR-128a, miR-389 and miR-9 suppresses dengue virus replication by promoting NF-kB–dependent IFN production. After 3 days’ post treatment with Acetaminophen, LRD and RDN, we carried out the GO enrichment and clustering analysis to find out the targets associated with the physiological features of dengue infections related to immune and inflammatory response.

### Immunity miRNAs expression profiling

**Fig. 2:**
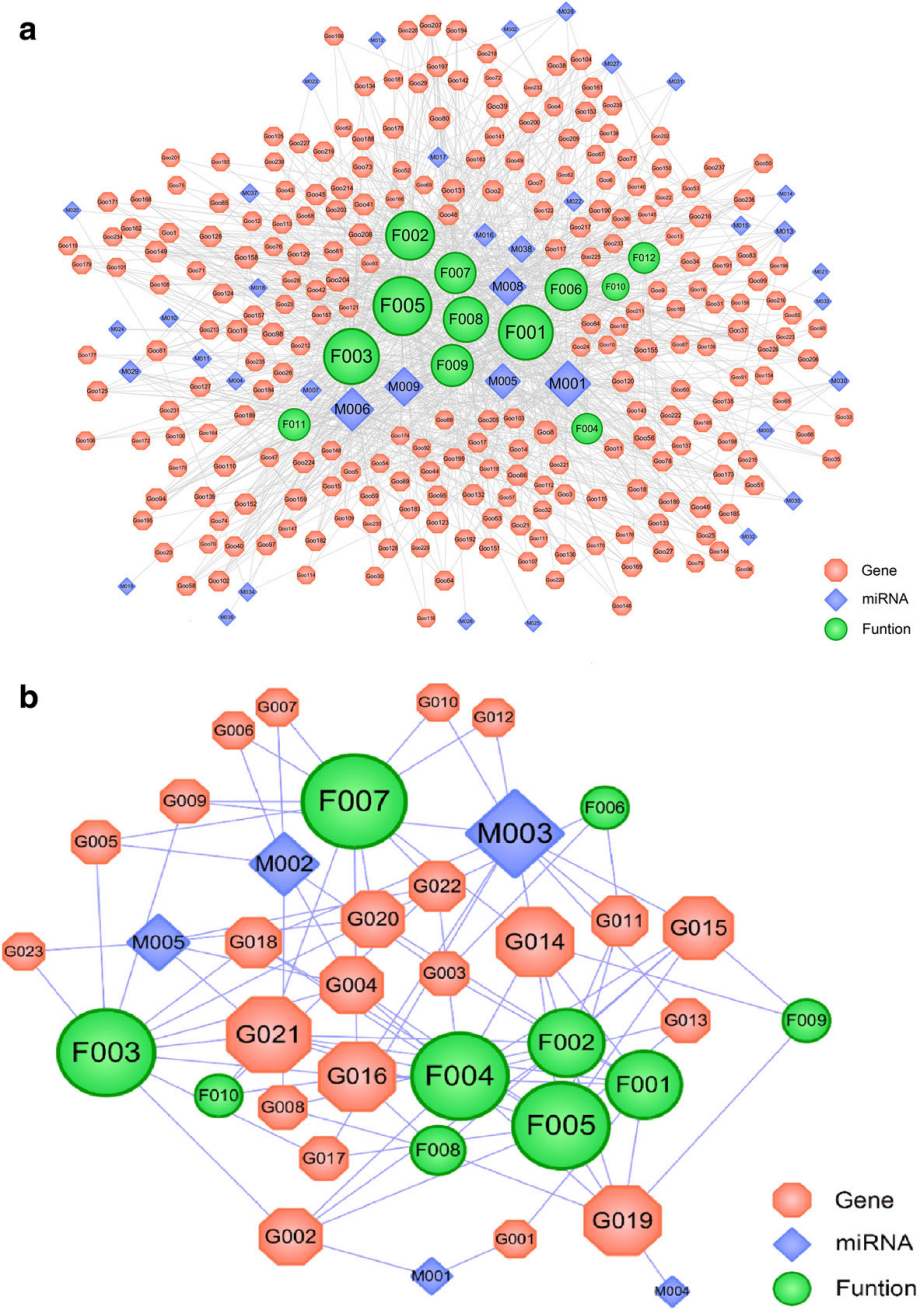
Network diagram of miRNAs-immune target-function pathway response to dengue virus (**a)** post treated by RDN and LRD drugs. **b** miRNAs-immune target-function pathway, response to dengue virus post treated by Acetaminophen drug. A miRNA and a target are linked if the target protein is hit by the corresponding miRNAs. Similarly, a target and a functional module are linked if the target is involved in this biological process. Node size is proportional to its degree and the letters are node labels

The integrative analysis of miRNAs expression recognizes a network inference of target immune response and functional interactions occurring in dengue infections patients. The result of miRNAs expression and reconstruction of a regulatory network in plasma-induced dengue symptoms post treatment by RDN with LRD are shown in Fig. 2a. The resultant Mi-T-P network which consisted of 38 differentially up-regulated expressed miRNAs and 239 integrative targets correlated with miRNAs, sorting from 2 to 64 degree and 44.4% of the genes being targeted by at least 2 miRNAs and their corresponding 12 functional annotations. The names of unique genes that are potential targets of miRNAs are PTEN (Phosphatase and Tensin Homolog) showing the highest degree (Degree = 12), RELA (Proto-Oncogene, NF-KB Subunit, Degree = 12), TRAF6 (TNF Receptor Associated Factor 6, Degree = 11) and IL6 (Interleukin 6, Degree = 11). Functional pathway analysis of anti-correlated target genes revealed various overrepresented biological processes including regulation of programmed cell death and apoptosis, regulation of cell proliferation, protein amino acid phosphorylation, immune response and response to cytokine stimulus with degree of 94, 85, 72, 57 and 55, respectively. The information of these parameters has been listed in the Additional file 5: Table S3.

With post-treated Acetaminophen medicine as a single therapy, there is a weak correlation between miRNAs expression and their targets. (Figure 2b) shows miRNAs differential expression in the supernatant of blood samples of patients. Among the up-regulated, M003 (miRNAs, hsa-miR-200c-3p) has the highest number of target candidate (degree = 10), followed by M002 (degree = 6), M005 (degree = 5), M001 (degree = 2) and M004 (degree = 1), indicating the one-target properties of ingredients. Results also showed 23 anticorrelated targets with the number of targets per miRNAs ranging from 2 to 8; and genes being targeted by one miRNA and their corresponding 12 functional annotations. The highest degree miRNAs are differently expressed compared with the patients treated with Acetaminophen therapy (regulation of programmed cell death and apoptosis, regulation of cell proliferation and protein amino acid phosphorylation with degree of 11, 10 and 10, respectively), which may be due to the effect of Acetaminophen on the expression of miRNAs in response to dengue virus. The detailed information of these miRNAs expressions, immune target genes and functional pathway post-treated with Acetaminophen are listed in the Additional file 6: Table S4.

Qi, et al. [41] Reported that cytokines and epigenetic regulators may be putative target genes of these miRNAs, and the pathway of biological regulation may be modulated by these miRNAs, miRNAs were classified as important ingredients of immune regulation [42]. Distinct miRNAs are capable of targeting genes that inhibit epigenetic pathways, while several miRNAs were confirmed to control down-regulation inflammatory responses by directly targeting genes encoding molecules [43, 44]. Additionally, during DENV2 infection miRNAs were differentially expressed, among these upregulated are (R-4290, −4279, −625*, −let-7e, −1290, −33a, −378, −1246, −767-5p, −320c, −720, −491-3p, −3647, −451 and −4286) but downregulated miRNA were (miR-106b,-20a, −30b and −3653) [41].

### Inflammatory miRNAs expression profiling

**Fig. 3:**
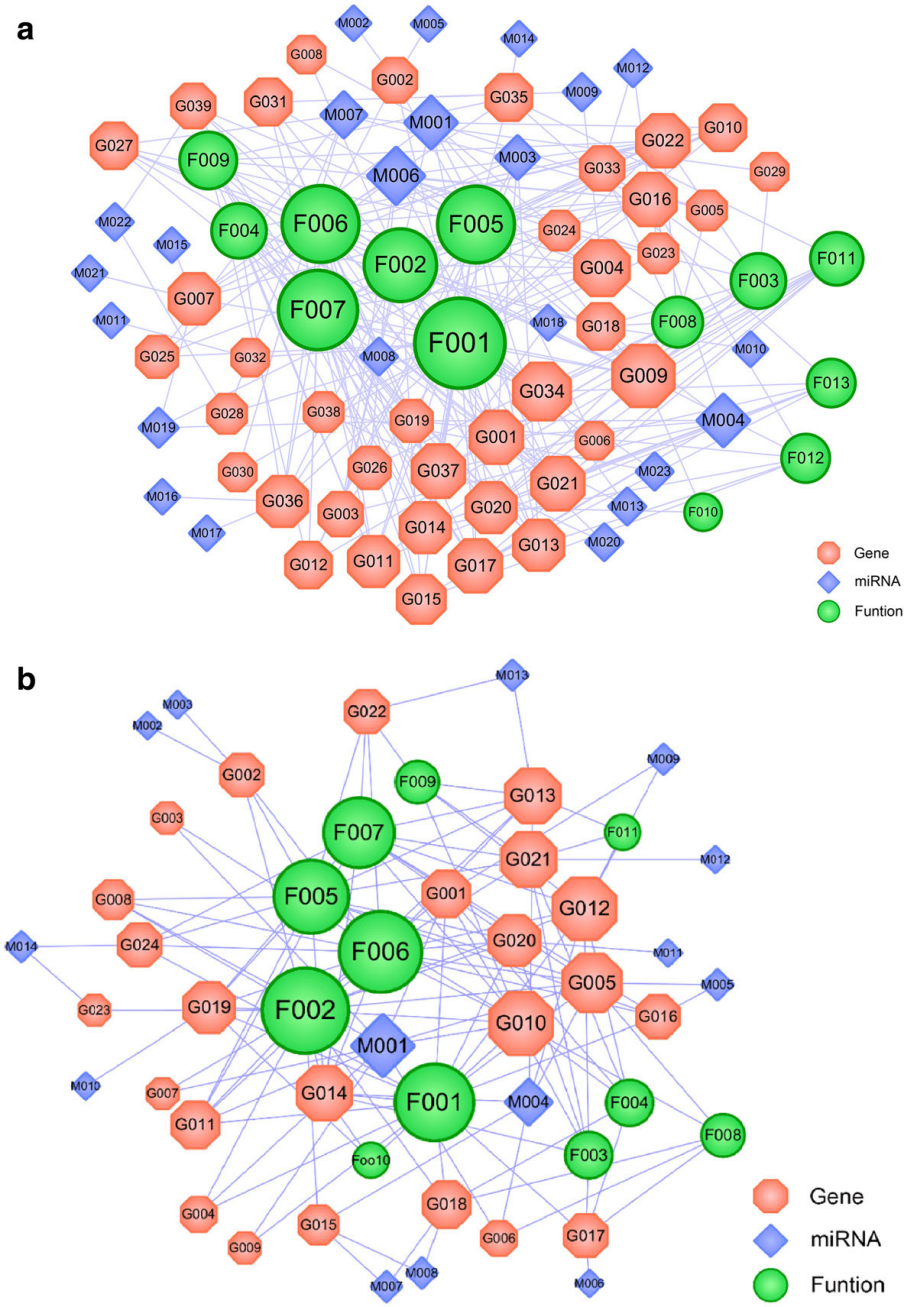
Network diagram of miRNAs-inflammatory target-function pathway relationship response to dengue virus (**a**) post treaded by RDN and LRD drugs. **b** miRNAs- inflammatory target-function pathway, response to dengue virus post treated by Acetaminophen drug. A miRNA and a target are linked if the target protein is hit by the corresponding miRNAs. Similarly, a target and a functional module are linked if the target is involved in this biological process. Node size is proportional to its degree and the letters are node labels

miRNAs expression induced dengue symptoms post treatment by RDN with LRD and (Fig. 3a) shows the interactions generated between 23 miRNAs and 39 predicted targets. The following action mode prediction showed that 15 of these miRNAs-target-pathway displayed up-regulation patterns, while the rest of them exhibited down-regulation patterns, and few of the targets are verified in previous research, such as VEGFA (Vascular Endothelial Growth Factors A) [45]. Through evaluate the molecular mechanism of RDN on MAPKs modulation, results in an activation of the mitogen-activated protein (MAP) kinase pathways, which in turn stimulates a variety of transcription factors such as NF-κB and AP-1, formerly definitely manages the production of pro-inflammatory cytokines, while downstream signaling effects that influence to inflammation [46]. Herbal medicine probably controls the infectious disease mainly based on immunomodulatory agents stimulation (such as GSK3B, MAPK14, PPARγ) will probably help innate and adaptive immune, and regulation the inflammatory cytokines and proinflammatory mediators (like IL-6, IL-8, TNF-α, COX2) [47]. More details are listed in the Additional file 7: Table S5).

In order to clarify the effect of Acetaminophen as anti-inflammatory therapy and to find the relationships between the active ingredients of miRNAs and their targets, the network was generated by connecting the potential miRNAs 14 and the potential targets 24 associated with the DENV. The mean number of potential targets per potential compound was 1.045. Among the 14 potential miRNAs, 2 have high degree distributions and each of them hits more than 10 and 6 potential targets (hsa-miR-107, hsa-miR-320d). All of the 24 targets showing down-regulations were linked by 2 until 10 miRNAs and some of them are verified in previous research (Fig. 3b). The information of these parameters has been listed in the Additional file 8: Table S6.

### Effect of antiviral inhibition on inflammatory immune response

**Fig. 4:**
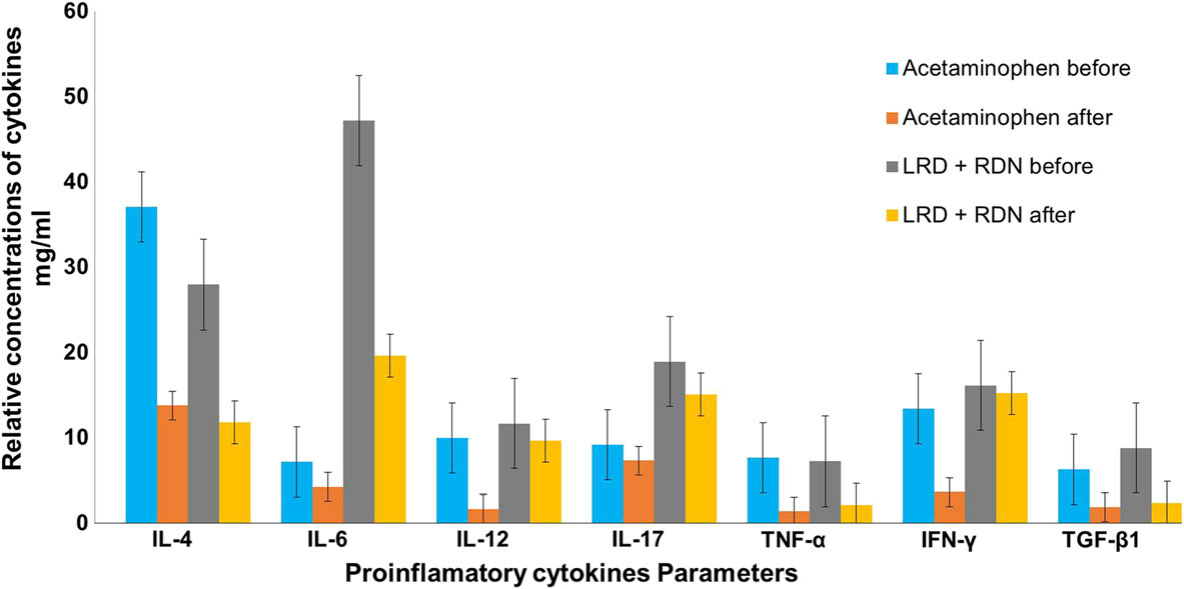
Acetaminophen, LRD and RDN induces proinflammatory cytokines production. Human supernatants infected with DENV in the presence or absence of Acetaminophen, LRD and RDN at infection and persisted in the medium. The clinical scores of patients treated with various doses, 1.3 g tablet orally three times daily of Acetaminophen or 10 mg of LRD tablet and 20 ml of RDN injection one time daily. IL-4, IL-6, IL-12, IL-17, TNF-α, IFN-γ, and TGF-β1 levels in the supernatants were measured by ELISA before treatment and 72 h post treatment. Data represent mean of three experiments

LRD and RDN inhibited DV-induced proinflammatory cytokines response parameters like TNF-α, IFN-γ, TGF-β1, IL-4, IL-6, IL-12, and IL-17 in a dose-dependent manner. That is the target of antiviral inhibition therapy to depress virus levels following treatment, toward relief symptoms of viral infection, like inflammation and haven, reducing the morbidity and the death-rate caused by the disease. Similar results were obtained in lung injuries using RDN drugs to inhibit the levels of IFN-γ and IL-6 [48], and RDN injection to significantly minimize the accumulations of TNF-α in sera and limb muscle tissues in mice [48]. Further, we examined whether the compound shown to reduce dengue immune parameters in blood were able to lower the inflammatory immune response in the patients. Cytokines parameters of all patients treated with Acetaminophen were inhibited after the treatment. Concomitant RDN with LRD, compared with Acetaminophen demonstrated greater inhibition in cytokines parameters (Fig. 4). However, cytokine concentration studies showed that concentrations of TNF-α, IFN-γ, TGF-β1, IL-4, IL-6, IL-12, and IL-17 were decreased in patients treated with RDN and LRD than in that treated with Acetaminophen (Fig. 4). These results indicate that the lowering of immune parameters in blood of patients by antiviral inhibition therapy may be decrease the intense inflammatory response caused by DENV and inflammatory cytokine concentrations in the patients’ blood. Induction levels of several cytokines, such as IFN-β, TNF-α, IL-6, MCP-1, and IL-1β in T-REx-293 cells were reported to be higher upon DENV-2 infection [49]. Whereas levels of IL-6, IL-10 and IFN-γ in mice treated with ribavirin or the combination therapy of ribavirin and RDN were all significantly decreased than the untreated group [50]. Our previous study confirmed an extremely decreased level of IL-1β, TNF-α and IL-6 parameters in RAW 264.7 cells treated with RDN and found that RDN may systematically disturb the NFL-κB and MAPK pathways. Therefore, RDN may block specific genes transcription partially through reducing the phosphorylation of IκB-α and the activation of NF-κB p65 [13].

### Influence of coagulation factor

**Fig. 5:**
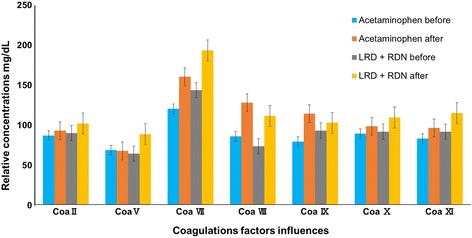
Anti-viral effects of continuous treatment with Acetaminophen, LRD and RDN were used to evaluate the anti-dengue virus activities of the LRD and RDN. Patients were treated with the LRD and RDN up to 3 days. The percentages of coagulants factors Coa II, Coa V, Coa VII, Coa VIII, Coa IX, Coa X and Coa XI, increased respectively after treatment, were obtained by comparing against before treated from triplicate experiments

This demonstrated that not only cytokines inhibited DF, but also human coagulants factor was used as a goal to minimize viremia through dengue infection. On behalf of the above feedback, we went ahead to study the impact of Acetaminophen given orally at a dose of 1.3 g three time daily for 3 days. Additionally, there was another treatment group with 20 ml RDN daily intravenous injections dose plus LRD 10 mg tablet each day. The present study showed significant lowering of viremia whereas increasing in the all coagulants factors Coa II, Coa V, Coa VII, Coa VIII, Coa IX, Coa X and Coa XI, respectively (Fig. 5) [51]. Recommended that, there is a correlation between activation of coagulation and clinical outcome of DENV infections. Some experimental tests have recommended that DENV and immunoglobulins from patients infected with the virus can directly impact the haemostatic regularity [52].

## Discussion

Treatment of DENV is presently a global infection and it is a viral disease for which there is no licensed therapeutic agent [53]. Treatment and diagnosis via new molecular-based techniques have become a new hope for early control, but are still exclusive because of their costs and normalization. DENV is the most important factors transmitted by mosquitoes worldwide and causing viral disease, almost 100 million cases of dangerous DENV each year, resulting in severe and life-threatening [54]. Direct anti-virus treatment that reduces the risk of dengue fever can be helpful although this may need to keep all serotypes effectively [55]. Predicting potential miRNA-disease associations created valuable contributions to promoting new drugs, explaining the pathogenesis concerning disorders and medication for distinct individual complex illnesses [16]. However, we have not completed a comprehensive outlook of all miRNAs expression regulation target genes through virus-human interactions in patients of DENV. A few human miRNAs might show antiviral miRNAs. When some delusive antiviral miRNAs were blocked by locked nucleic acid-modified antisense oligoribonucleotides, the hosts miss carriage to suppress viral replication [56]. Otherwise, in few cases, human miRNAs expression might be corroborated by viruses to reshape the human intracellular medium to benefit viral replication [57].

In the present study, results showed that about 89 human miRNAs are important in the DENV interactions before medication, which are of interest in human immune and inflammatory responses. Therefore, our study provides the most human miRNAs and their predicted functions pathways mediated by them in response to virus infection. In contrast, similar studies in mammals, revealed that the miRNAs profiles were reshaped by hepatitis C virus and miRNAs of human might have been correlated with the regulation of human immune systems [58]. Accordingly, virus contagion has a capability of destructing processions of cellular functions of the host on several levels, like the effects of the cell cycle or apoptosis of cells infected with viruses, including miRNAs [59]. Through cellular, miRNAs of virus-host interactions play a key regulators of gene expression [60]. Further experiments concluded that the regulation of Ad6 by miR-122 could significantly improve the safety profile of the entire body rearward systemic administration. Consequently, also recognizing disease-related miRNAs could adequately improve disease biomarker detection for the medication, it is necessary to develop robust computational models to predict innovative human disease-miRNA associations [15, 61].

Microarray analysis in the present study displayed that hsa-miR-320a; hsa-miR-107 and hsa-miR-361-5p were extremely preserved in human. In addition, analyses predicted that miR-320a; one of the miRNAs highly preserved could target the Mitogen-Activated Phosphatase and Tensin Homolog (PTE), and DEAD-Box Helicase 3, X-Linked (DDX3X). Recent studies revealed that PTEN and DDX3X were activated by invading DENV, suggesting that host might inhibit virus infection by targeting viral transcripts with host miRNAs [62]. To reduce the dengue fever impact using a potential anti-dengue medicine, we administered several known antiviral drugs (Acetaminophen, LRD and RDN), oral treatment or injection targeting DENV were found to significantly reduce viremia. Additionally, LRD combination with RDN showed a significant reduction of viremia and indicated that RDN inhibitors may be interesting drug candidates for DENV. They also have a potential for antiviral efficacy in vivo, similar to their proposed used in anti–hepatitis C [63], whereas Acetaminophen treatment may have little effect during dengue fever treatment.

Furthermore the potential of RDN and LRD against DENV in patient are bitten by carrier mosquitoes. The results showed that RDN could reduce the immune parameters in blood of patients if mixed with LRD tablet. The suggested mechanism was that RDN down-regulated NF-B activation reducing the expression of pro-inflammatory cytokines and increasing IFITM3 expression via MAVS, giving a sign that the anti-virus effects of RDN were related to adjusting the unbalanced homeostasis of the body caused by stress response, but unrelated with direct anti-virus activity [64, 65] Reported that RDN could inhibit the susceptibility of influenza in restraint-stressed mice, through RDN down-regulating NF-kB activation to constrict the expression of pro-inflammatory cytokines and enhance IFITM3 mRNA expression via MAVS. This outline suggested that the anti-influenza effects of RDN were regarding homeostasis of the body caused by stress response, however not concerning with direct anti-virus activity. RDN injection has various functions; such as absolution heat, eliminate flatus, and detoxification, clinically used in the curative of hyperpyrexia, influenza and body pain, cough, yellow sputum, and other symptoms raised by respiratory tract infection. RDN injection has been openly applied with good clinical performance [11].

Until now, it has been cloudy how dengue viremia levels contribute to the severity of the disease, after that reducing viremia by antiviral drugs to reduce the symptoms of DF [66]. Found that higher level of viremia was linked with increased DENV intensity and proposed that lowering viremia may reduce morbidity and the risk of dengue hemorrhagic fever or dengue shock syndrome. Infected mice by dengue showing increased TNF-a, IFN-g and IL-6 levels, which were recognized as triggers for DENV were also reported. We have found that reducing blood some immune parameters through the use of antiviral treatment significantly decreases proinflammatory cytokine levels and increases the concentrations of coagulants factors in the blood of treated patients. These results indicated that lowering proinflammatory cytokine parameters through timely antiviral drug treatment may improve the severity of DF symptoms and perhaps decrease the risk of progression.

Finally, this work suggests ways to prohibit the gaining of DENV which is to avoid being bitten by mosquitoes that carries the disease. Although this can be done by avoiding areas where dengue is endemic, but may be that is not the ideal strategy because it would require a person to avoid almost of the tropical and subtropical areas of the world, many of which are popular purpose to travel, work or at least using natural repellent for mosquitoes.

## Conclusions

Based on the results of this study, it was concluded that RDN could be used as anti-dengue in vivo, to reduce the susceptibility and severity of dengue virus. This study also warrants the future in vivo anti-viral as part of the developmental process for development RDN as potential anti-dengue therapeutic. We therefore, need more investigations and specific diagnostic criteria. However, this study with a few number of sample size, gives a promising opportunity in the treatment of DENV using RDN in combination with LRD.

## Additional files

Additional file 1: Differentially expressed miRNAs and Correlation coefficient matrix. (XLSX 64 kb)

Additional file 2: Volcano plots showing differentially expressed miRNAs in human supernatants infected with DENV in the presence or absence of Acetaminophen, LRD and RDN at infection and persisted in the medium. (DOCX 473 kb)

Additional file 3: Table S1. MiRNAs and predicted target gene response to dengue virus infection. (DOCX 61 kb)

Additional file 4: Table S2. MiRNAs, target gene and function pathway process response to dengue virus infection. (DOCX 56 kb)

Additional file 5: Table S3. MiRNAs, Immune target gene and function pathway process response to dengue virus treated by RDN with LRD. (DOCX 48 kb)

Additional file 6: Table S4. MiRNAs, immune target gene and function pathway process response to dengue virus treated by Acetaminophen. (DOCX 27 kb)

Additional file 7: Table S5. MiRNAs, inflammatory target gene and function pathway process response to dengue virus treated by RDN with LRD. (DOCX 29 kb)

Additional file 8: Table S6. MiRNAs, inflammatory target gene and function pathway process response to dengue virus treated by Acetaminophen. (DOCX 27 kb)
