# Correction to: dengue virus causes changes of MicroRNA-genes regulatory network revealing potential targets for antiviral drugs

**DOI:** 10.1186/s12918-018-0536-3

**Published:** 2018-02-23

**Authors:** Mohamed Shahen, Zihu Guo, Akhtar Hussain Shar, Reham Ebaid, Qin Tao, Wenjuan Zhang, Ziyin Wu, Yaofei Bai, Yingxue Fu, Chunli Zheng, He Wang, Piar Ali Shar, Jianling Liu, Zhenzhong Wang, Wei Xiao, Yonghua Wang

**Affiliations:** 1 College of Life Science, Northwest A & F University, Yangling, Shaanxi 712100 China; 2 Center of Bioinformatics, Northwest A & F University, Yangling, Shaanxi 712100 China; 3 Zoology Department, Faculty of Science, Tanta University, Tanta, 31527 Egypt; 4 School of Environment and Safety Engineering, Jiangsu University, Zhenjiang, Jiangsu 212013 China; 5 College of Life Science, Northwest University, Xi’an, Shaanxi 710069 China; 6 State Key Laboratory of New-tech for Chinese Medicine Pharmaceutical Process, Lianyungang, Jiangsu 222001 China

## Correction

After publication of the article [1], it has been brought to our attention that an author’s name was spelt incorrectly in the original published article. Yonghua Wang was previously spelt “Yonghua Wan”. This has now been corrected in the revised version of the article.
