# Supplementary material for: Dengue virus causes changes of MicroRNA-genes regulatory network revealing potential targets for antiviral drugs
Source: BMC Syst Biol. 2018 Jan 4;12:2. doi: 10.1186/s12918-017-0518-x (PMC5753465; doi:10.1186/s12918-017-0518-x)

**Dengue virus causes Changes of MicroRNA-Genes Regulatory Network revealing potential Targets for Antiviral Drugs.**

Volcano plots showing differentially expressed miRNAs in human supernatants infected with DENV in the presence or absence of Acetaminophen, LRD and RDN at infection and persisted in the medium.


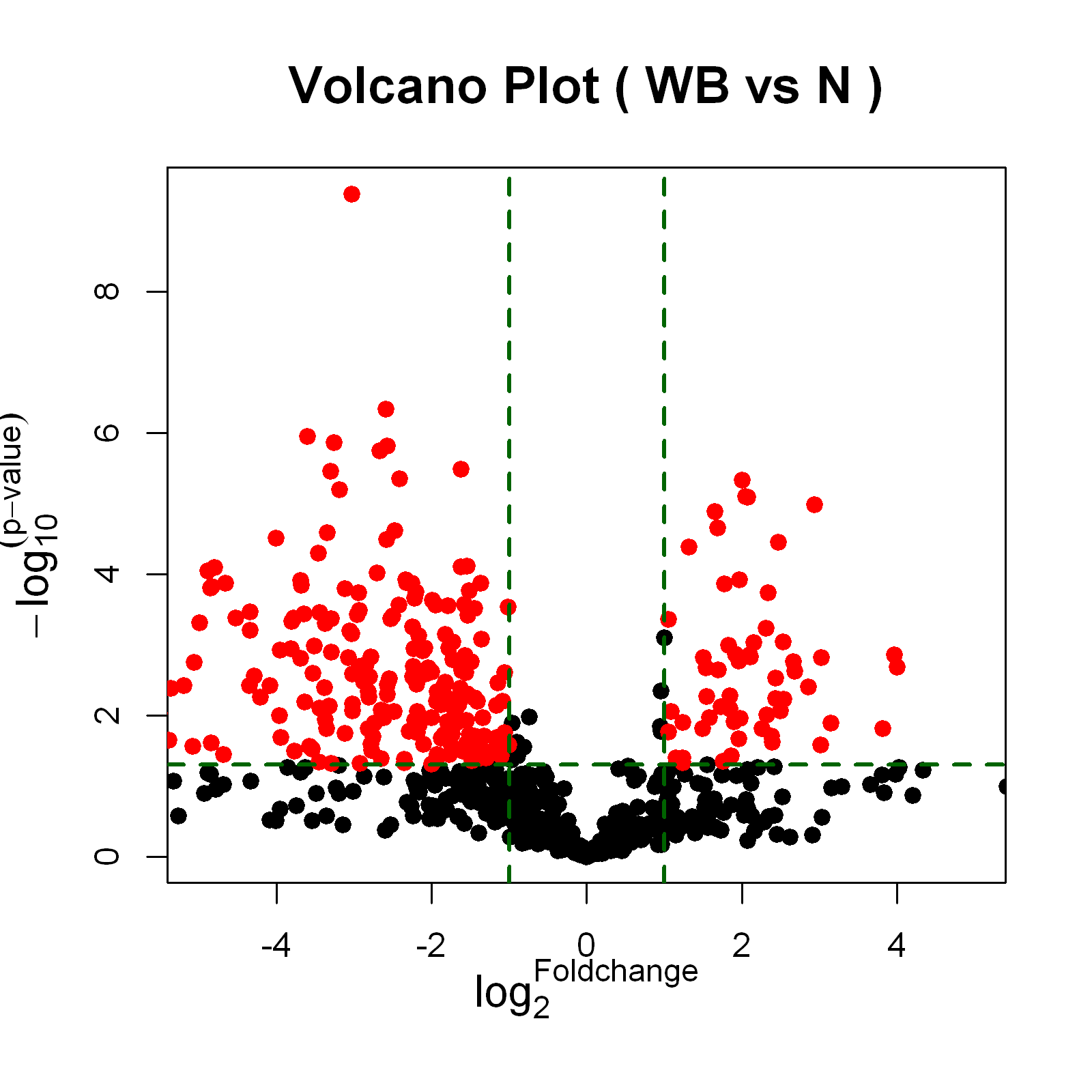

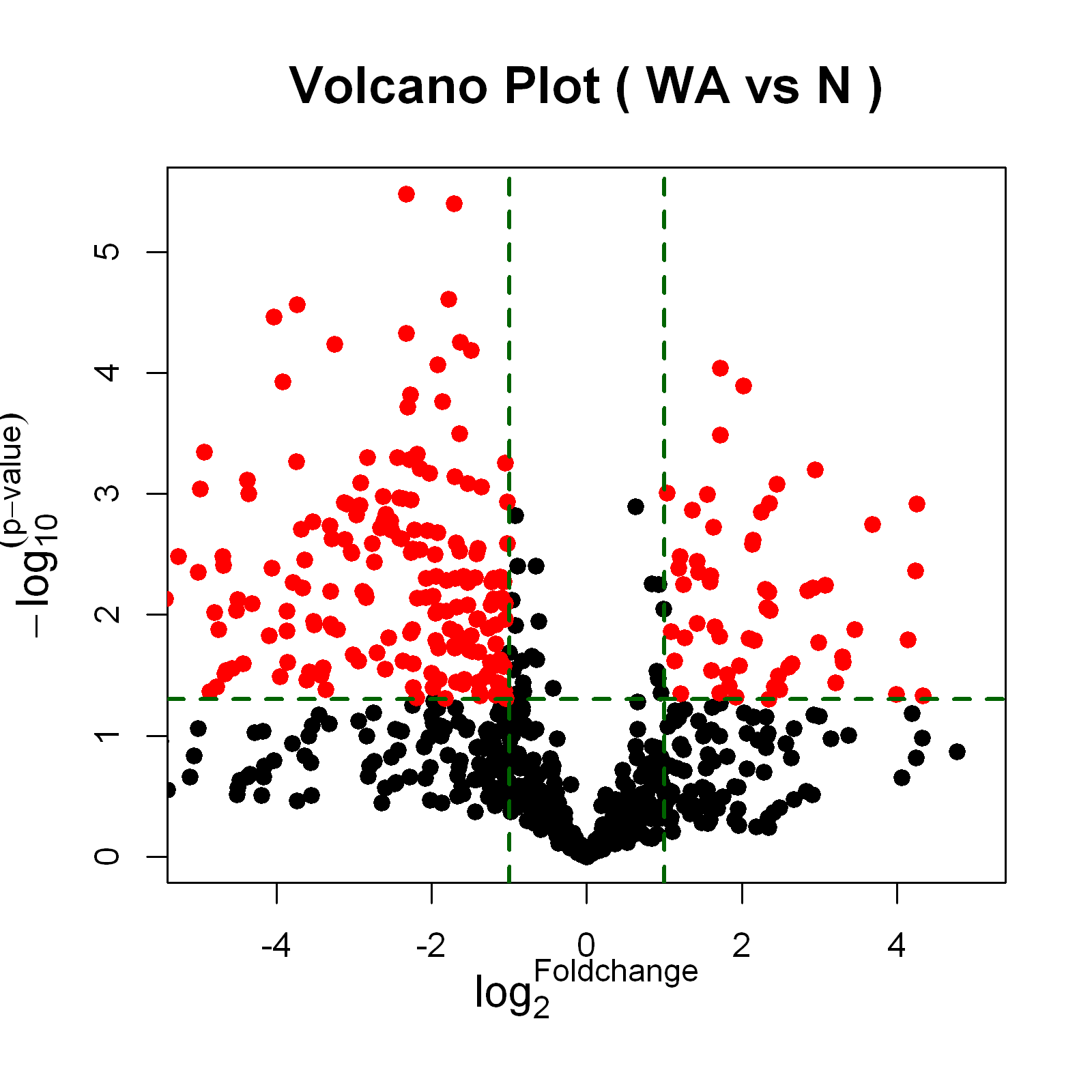

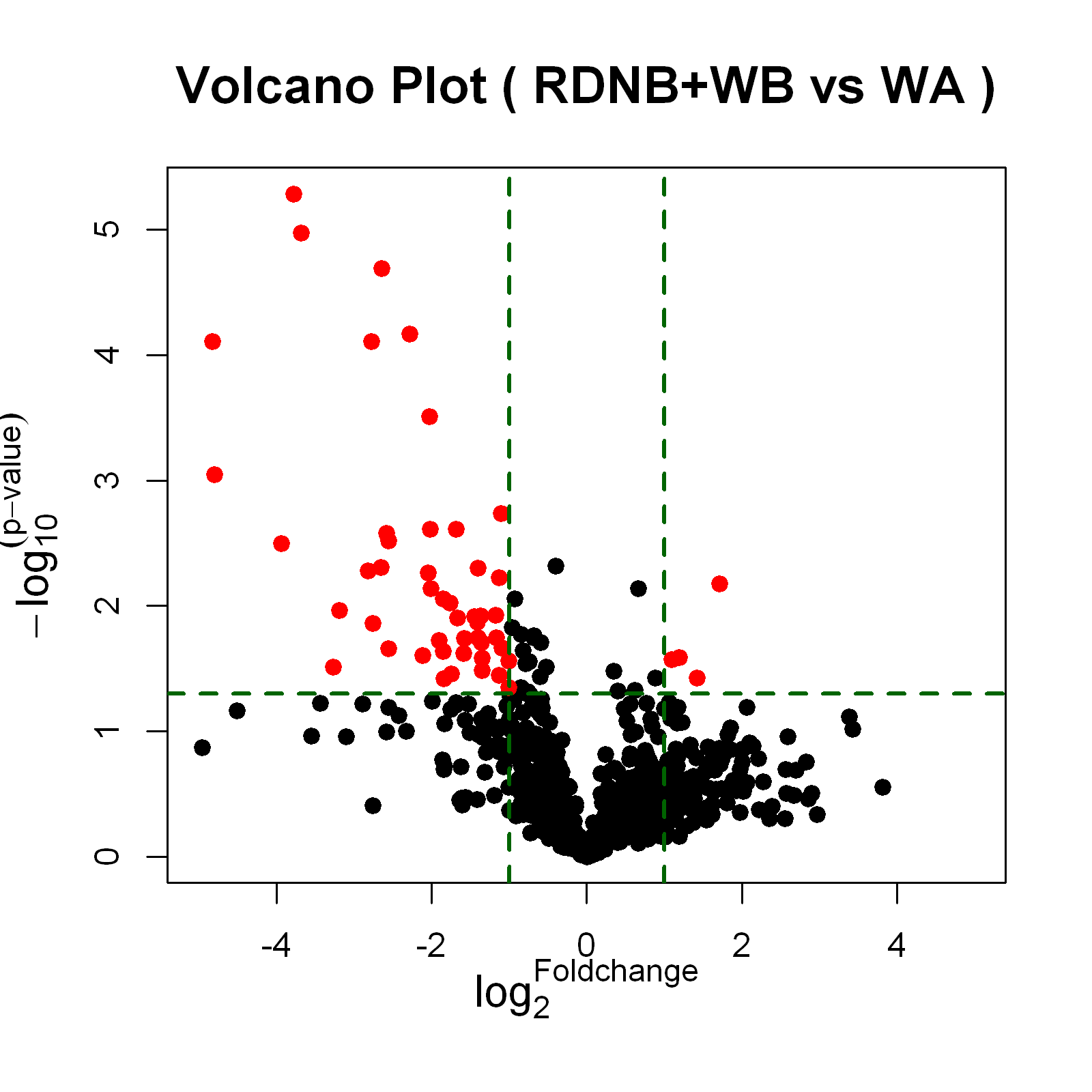

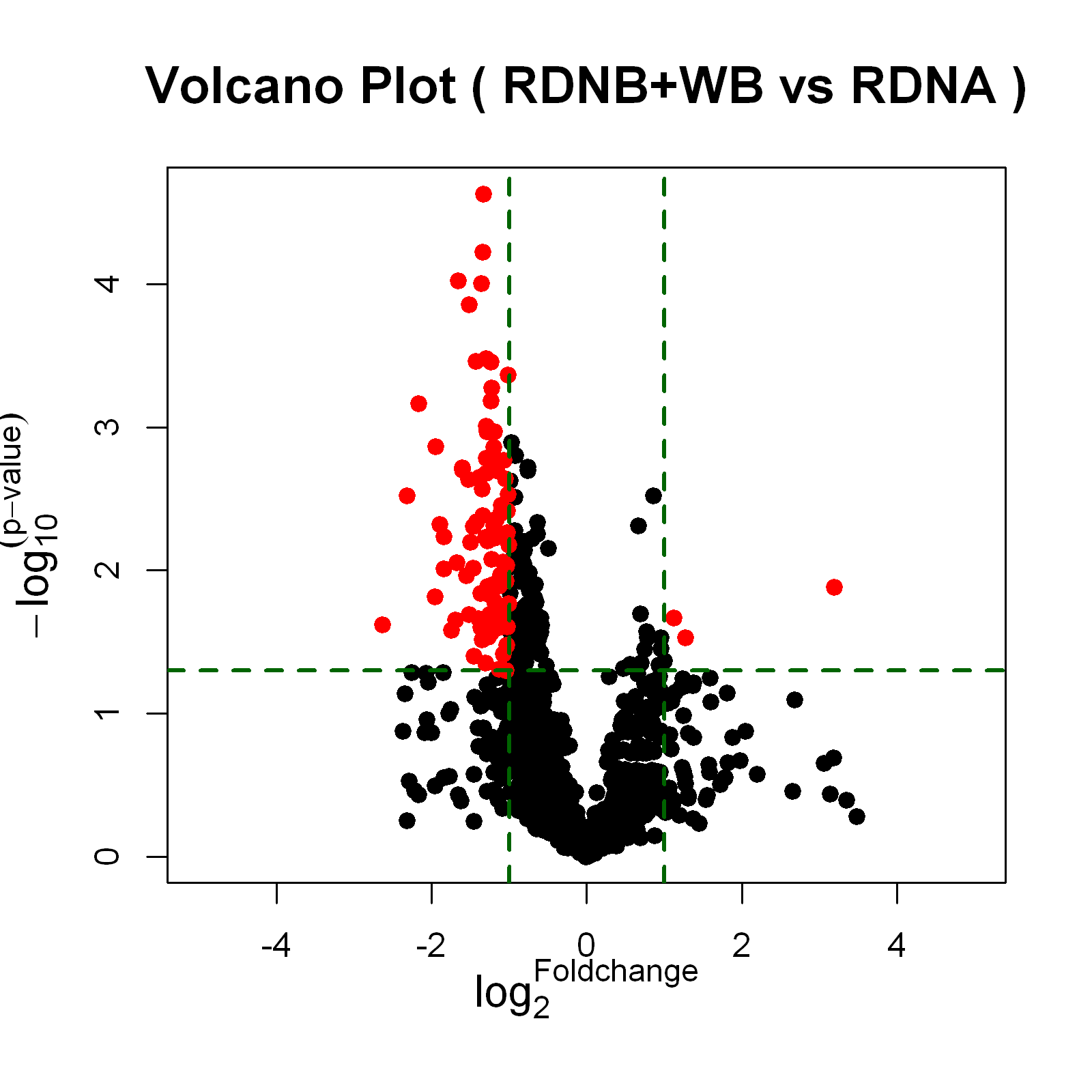

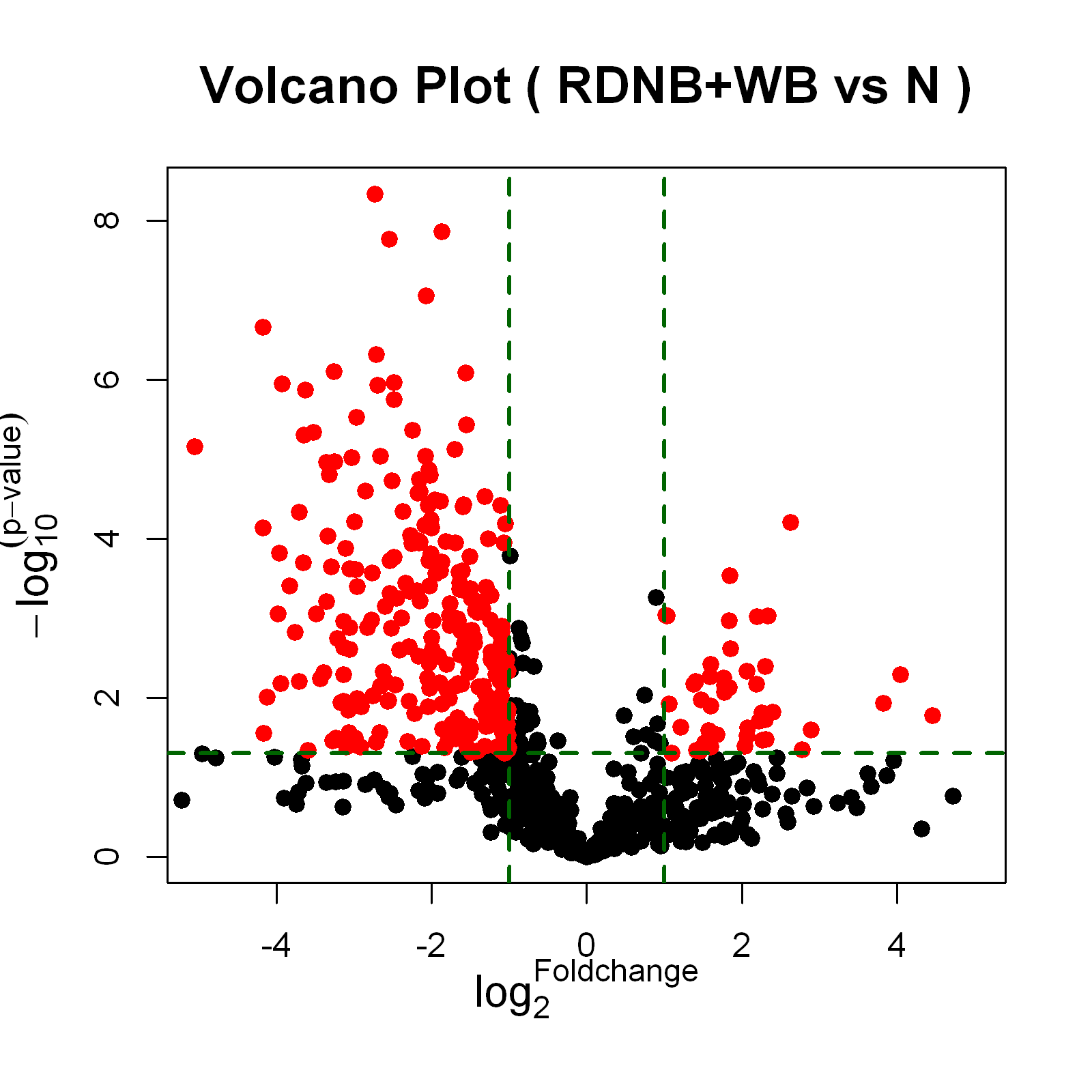

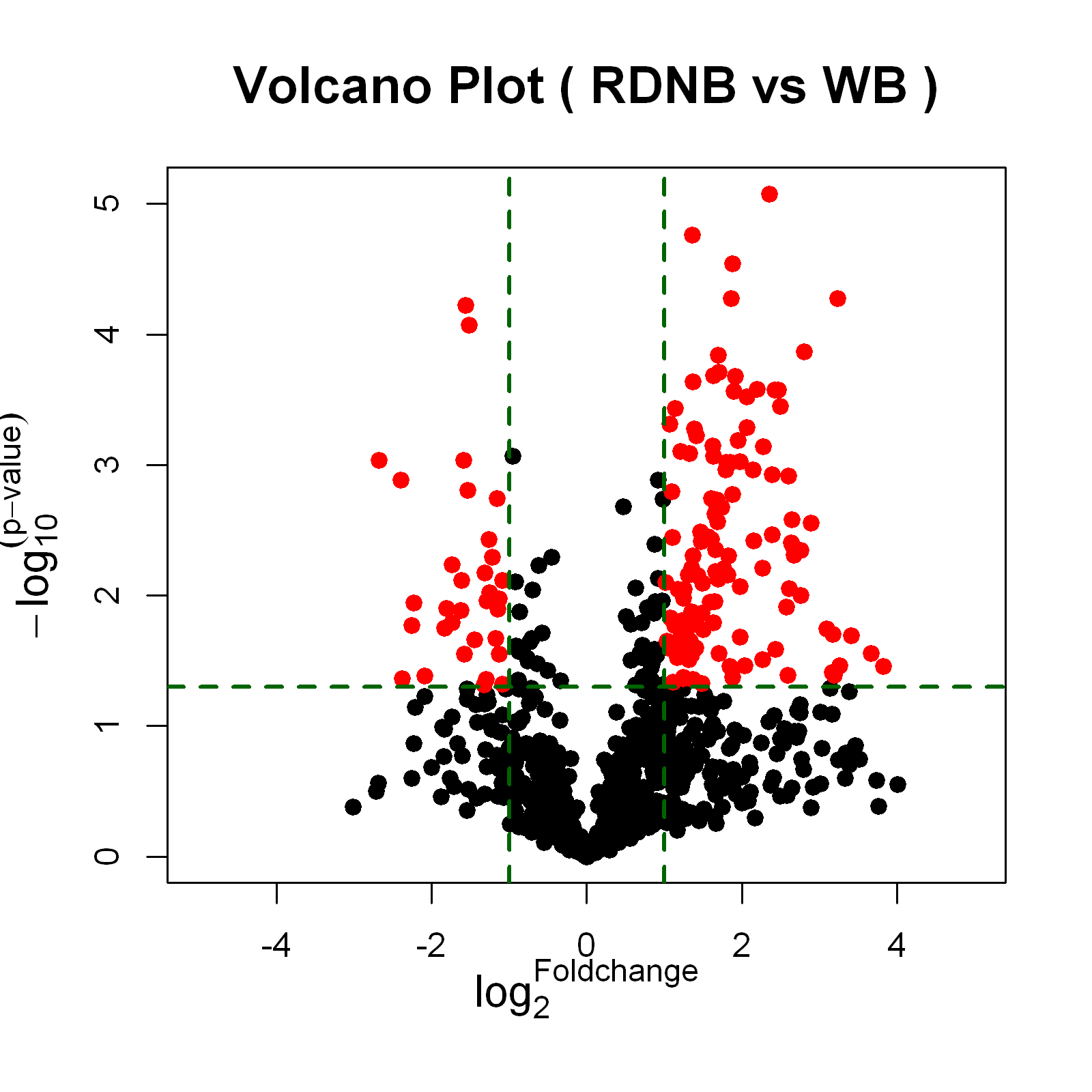

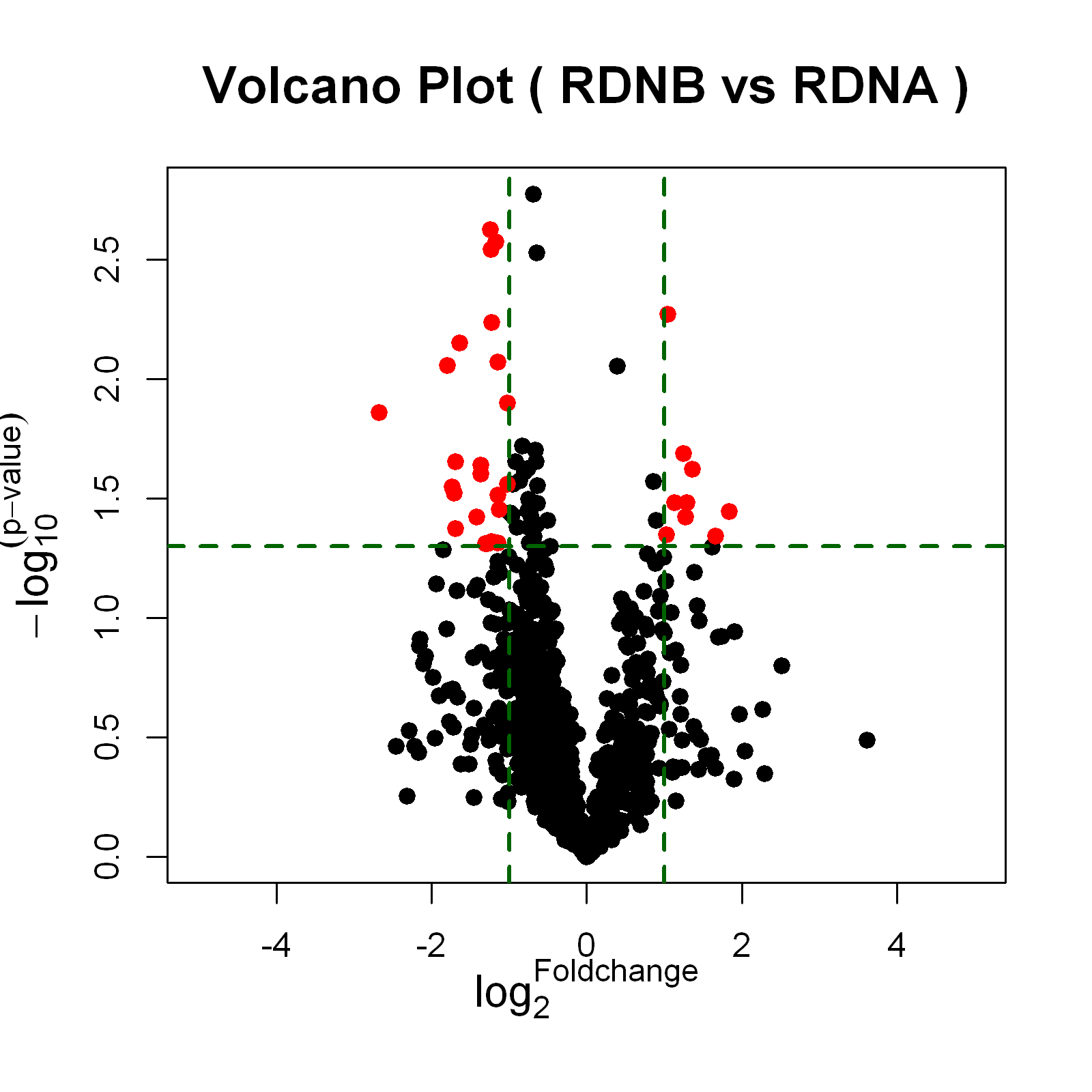

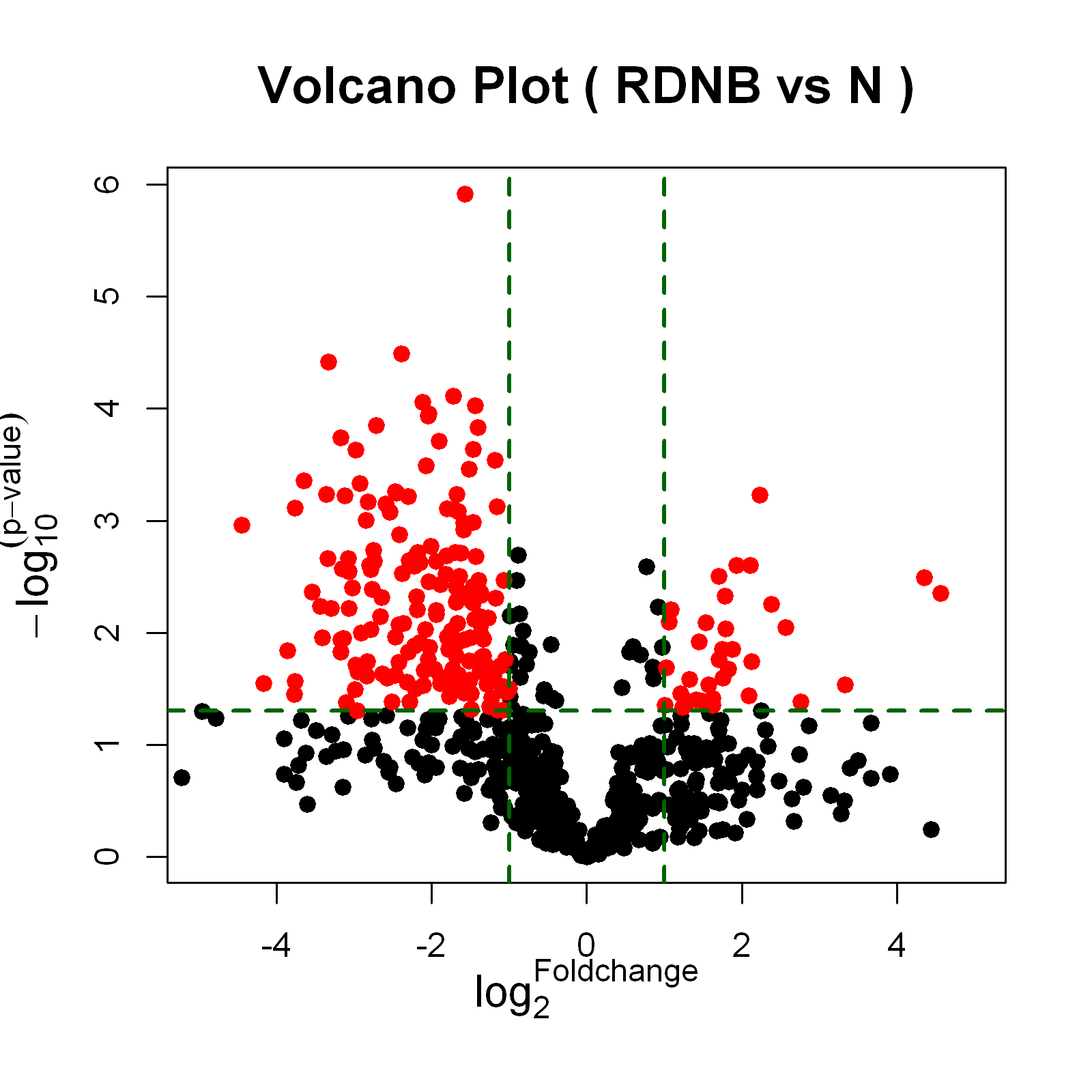

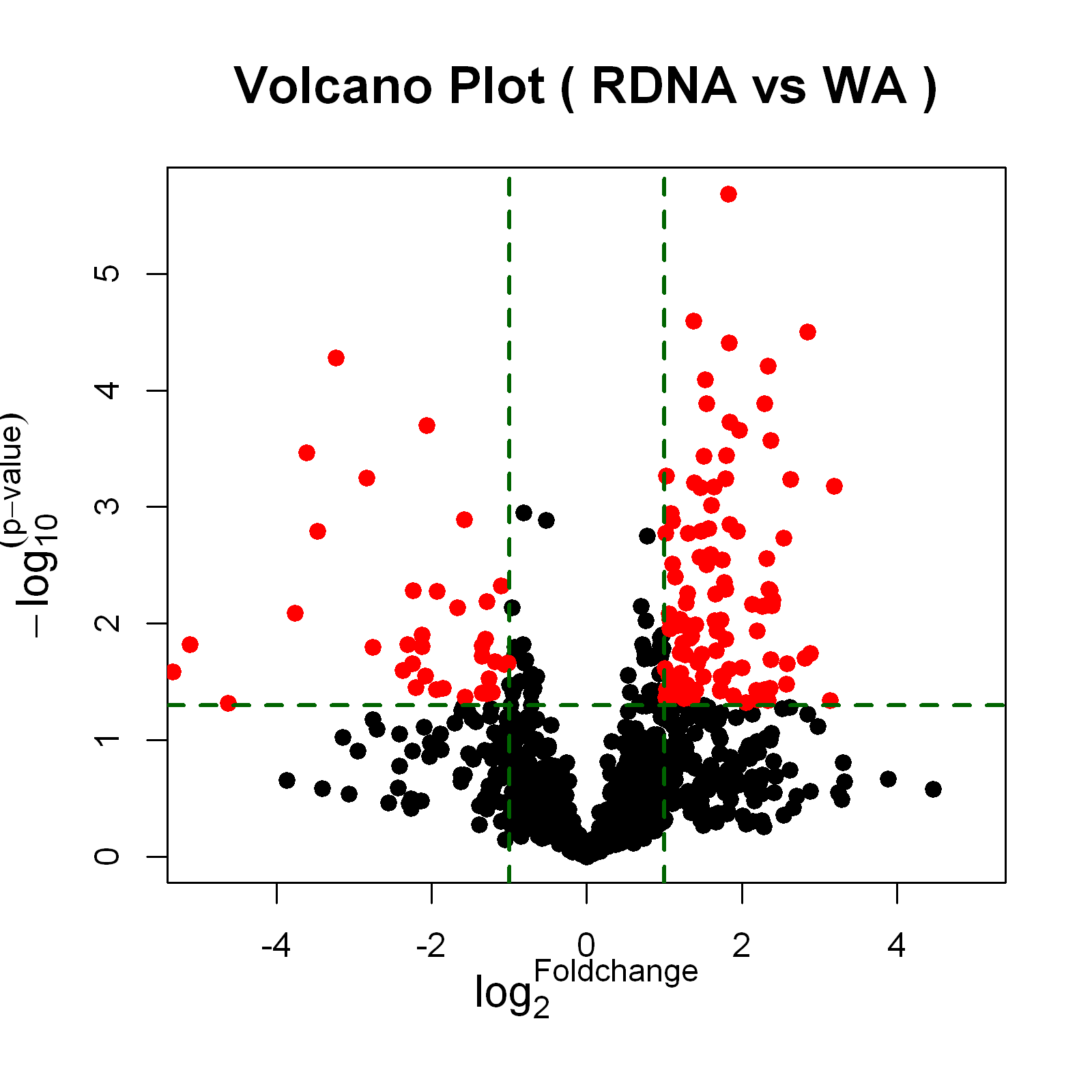

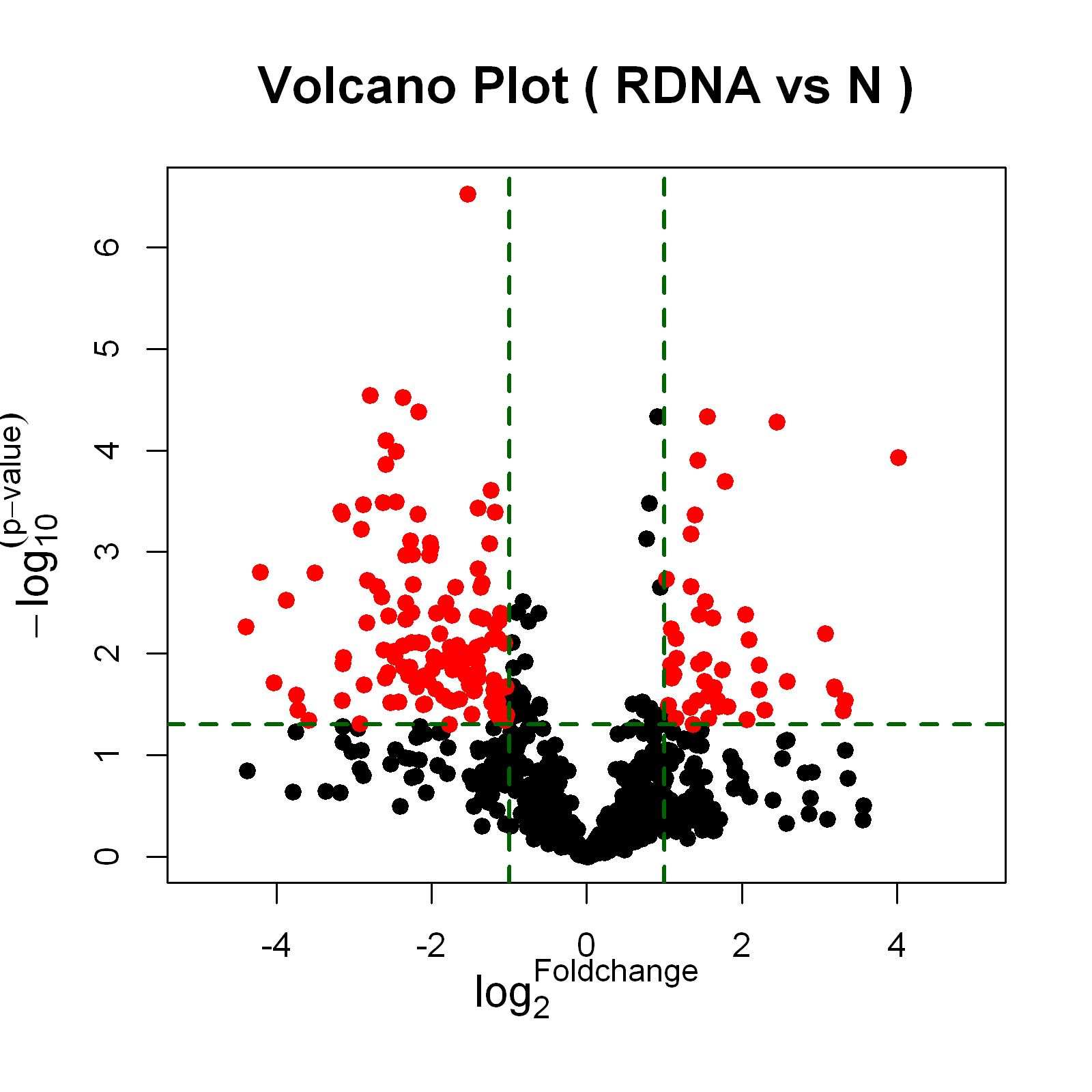

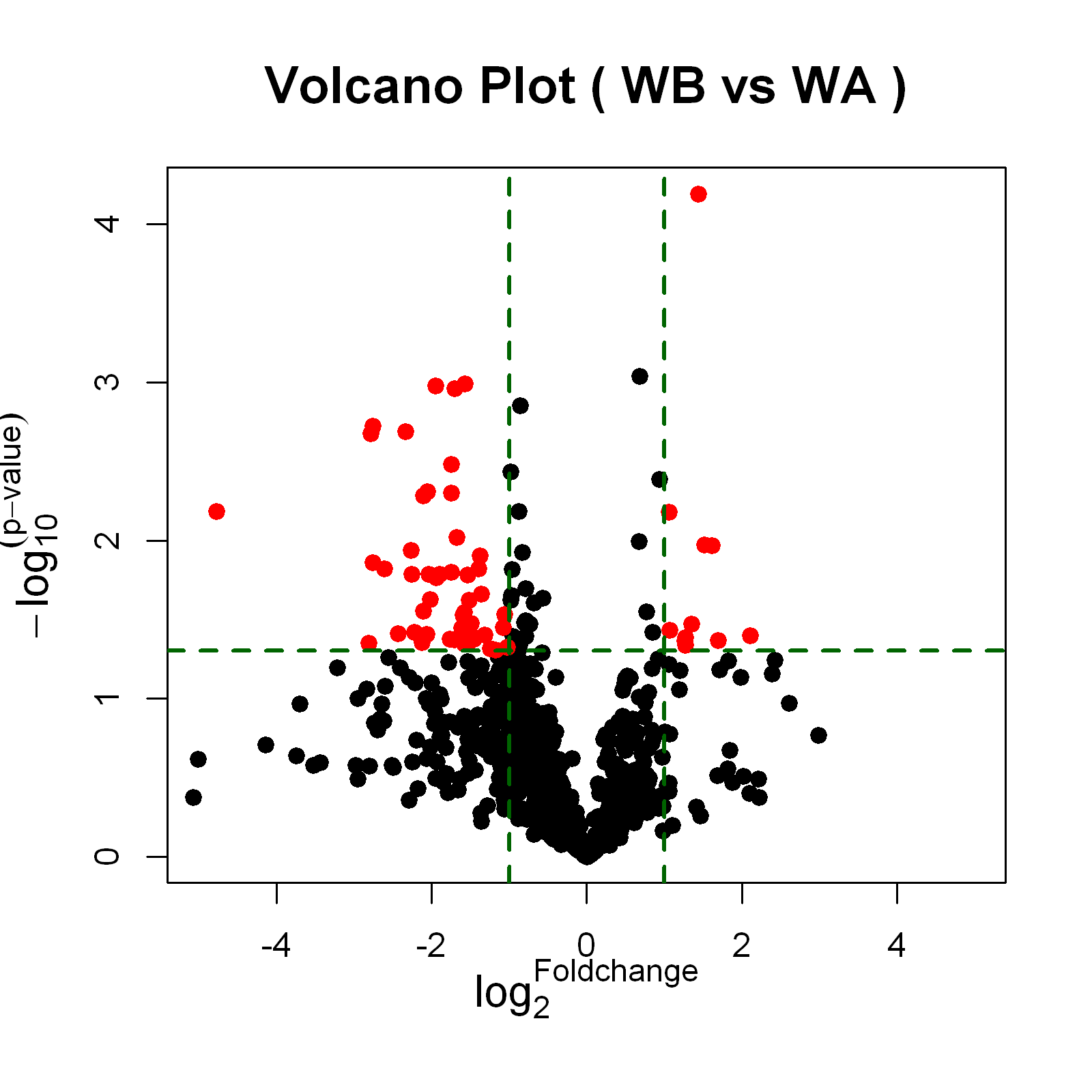

Supplement: Supplementary file 2 — Volcano plots showing differentially expressed miRNAs in human supernatants infected with DENV in the presence or absence of Acetaminophen, LRD and RDN at infection and persisted in the medium. (DOCX 473 kb) [file 12918_2017_518_MOESM2_ESM.docx]
