# Supplementary material for: Dengue virus causes changes of MicroRNA-genes regulatory network revealing potential targets for antiviral drugs
Source: BMC Syst Biol. 2018 Jan 4;12:2. doi: 10.1186/s12918-017-0518-x (PMC5753465; doi:10.1186/s12918-017-0518-x)
Supplement: Supplementary file 3 — MiRNAs and predicted target gene response to dengue virus infection. (DOCX 61 kb) [file 12918_2017_518_MOESM3_ESM.docx]

**Dengue virus causes Changes of MicroRNA-Genes Regulatory Network revealing potential Targets for Antiviral Drugs.**

**Table S1**

MiRNAs and predicted target gene response to dengue virus infection.

| **Gene symbol** | **ID** | **Degree** | **miRNAs Name** | **ID** | **Degree** |
| --- | --- | --- | --- | --- | --- |
| DDX3X | G1481 | 18 | hsa-miR-320a | M005 | 134 |
| PTEN | G1496 | 17 | hsa-miR-107 | M002 | 102 |
| RORA | G1447 | 15 | hsa-miR-361-5p | M013 | 93 |
| PPP3R1 | G1392 | 14 | hsa-miR-542-3p | M017 | 73 |
| VEGFA | G1466 | 13 | hsa-miR-146b-5p | M015 | 70 |
| DICER1 | G1331 | 12 | hsa-miR-142-3p | M010 | 67 |
| ADRB2 | G1465 | 12 | hsa-miR-876-5p | M018 | 67 |
| ZFPM2 | G1410 | 12 | hsa-miR-140-5p | M009 | 58 |
| RICTOR | G1457 | 11 | hsa-miR-125a-5p | M011 | 52 |
| S1PR1 | G1235 | 11 | hsa-miR-375 | M003 | 51 |
| IGF1R | G1452 | 10 | hsa-miR-455-5p | M016 | 44 |
| FXR1 | G1230 | 10 | hsa-miR-193a-3p | M012 | 39 |
| ERBB2IP | G1297 | 10 | hsa-miR-106b-5p | M028 | 28 |
| RAD21 | G1493 | 10 | hsa-miR-106a-5p | M027 | 27 |
| KAT2B | G1484 | 10 | hsa-miR-19a-3p | M054 | 20 |
| PRDM1 | G1423 | 10 | hsa-miR-19b-3p | M055 | 20 |
| ANKRD17 | G1417 | 10 | hsa-miR-324-5p | M014 | 19 |
| NRAS | G1456 | 10 | hsa-miR-130b-3p | M033 | 18 |
| KLF4 | G1482 | 9 | hsa-miR-130a-3p | M032 | 17 |
| CCDC88A | G1234 | 9 | hsa-miR-25-3p | M067 | 16 |
| TSC1 | G1223 | 9 | hsa-miR-30c-5p | M071 | 16 |
| ITPR1 | G1492 | 9 | hsa-miR-4500 | M082 | 16 |
| PRKCE | G1494 | 9 | hsa-miR-92a-3p | M088 | 16 |
| ADAM10 | G1490 | 9 | hsa-miR-181a-5p | M044 | 15 |
| ATG16L1 | G1232 | 9 | hsa-miR-30a-5p | M070 | 15 |
| ATG5 | G1382 | 9 | hsa-miR-30d-5p | M072 | 15 |
| SOCS6 | G1434 | 9 | hsa-miR-181b-5p | M045 | 14 |
| MAP3K1 | G1462 | 9 | hsa-miR-181c-5p | M046 | 14 |
| ELMOD2 | G1383 | 9 | hsa-miR-181d-5p | M047 | 14 |
| HIF1AN | G1458 | 9 | hsa-miR-23c | M065 | 14 |
| NFAT5 | G1413 | 9 | hsa-miR-101-3p | M025 | 13 |
| HIF1A | G981 | 8 | hsa-miR-124-3p | M030 | 13 |
| SOCS5 | G1491 | 8 | hsa-miR-15b-5p | M043 | 12 |
| SIAH1 | G1436 | 8 | hsa-miR-23a-3p | M063 | 12 |
| NRIP1 | G1394 | 8 | hsa-miR-23b-3p | M064 | 12 |
| TMED7 | G1291 | 7 | hsa-miR-302a-3p | M069 | 12 |
| CTNND1 | G1473 | 7 | hsa-miR-152-3p | M041 | 11 |
| RAB11A | G1339 | 7 | hsa-miR-15a-5p | M042 | 11 |
| BECN1 | G1395 | 7 | hsa-miR-186-5p | M049 | 11 |
| TNFAIP3 | G1303 | 7 | hsa-miR-29a-3p | M068 | 11 |
| SNX27 | G1239 | 7 | hsa-miR-424-5p | M079 | 11 |
| RB1 | G1476 | 7 | hsa-miR-148b-3p | M039 | 10 |
| USP2 | G1386 | 7 | hsa-miR-1260b | M008 | 8 |
| CASP7 | G1295 | 7 | hsa-miR-223-3p | M062 | 8 |
| MAP2K1 | G1163 | 7 | hsa-miR-374a-5p | M078 | 8 |
| GATA6 | G1188 | 6 | hsa-let-7f-5p | M022 | 7 |
| ITGB1 | G1166 | 6 | hsa-miR-145-5p | M037 | 7 |
| MFN2 | G986 | 6 | hsa-miR-24-3p | M066 | 7 |
| ARF6 | G1157 | 6 | hsa-miR-340-5p | M076 | 7 |
| CAPRIN1 | G774 | 6 | hsa-miR-365a-3p | M077 | 7 |
| TCF4 | G781 | 6 | hsa-miR-4306 | M081 | 7 |
| TRIB2 | G854 | 6 | hsa-miR-451a | M001 | 6 |
| VLDLR | G1345 | 6 | hsa-let-7b-5p | M019 | 6 |
| ABCA1 | G1405 | 6 | hsa-let-7c-5p | M020 | 6 |
| IL1A | G1165 | 6 | hsa-let-7d-5p | M021 | 6 |
| LGR4 | G1453 | 6 | hsa-let-7i-5p | M023 | 6 |
| RAC1 | G866 | 6 | hsa-miR-183-5p | M048 | 6 |
| TXNIP | G1341 | 6 | hsa-miR-197-3p | M053 | 6 |
| CLTC | G1240 | 6 | hsa-miR-7-5p | M086 | 6 |
| PPARGC1A | G1298 | 6 | hsa-miR-138-5p | M035 | 5 |
| MAPK9 | G1432 | 6 | hsa-miR-21-5p | M057 | 5 |
| SAMHD1 | G1366 | 6 | hsa-miR-31-5p | M073 | 5 |
| BCL11B | G1488 | 6 | hsa-miR-935 | M007 | 4 |
| JAK1 | G1385 | 6 | hsa-miR-103a-3p | M026 | 4 |
| CHUK | G1293 | 6 | hsa-miR-196a-5p | M052 | 4 |
| EDN1 | G1460 | 6 | hsa-miR-205-5p | M056 | 4 |
| MAP3K12 | G1218 | 6 | hsa-miR-22-3p | M059 | 4 |
| PIK3CB | G1486 | 6 | hsa-miR-125b-5p | M031 | 3 |
| NFATC3 | G1468 | 6 | hsa-miR-146a-5p | M038 | 3 |
| CDK6 | G1404 | 5 | hsa-miR-190a-5p | M050 | 3 |
| CRKL | G1055 | 5 | hsa-miR-221-3p | M060 | 3 |
| BCL2 | G834 | 5 | hsa-miR-222-3p | M061 | 3 |
| MAPK1 | G1181 | 5 | hsa-miR-425-5p | M080 | 3 |
| MAP2K4 | G1495 | 5 | hsa-miR-4644 | M083 | 3 |
| NUMB | G1396 | 5 | hsa-miR-513a-5p | M085 | 3 |
| RELA | G1474 | 5 | hsa-miR-10a-5p | M029 | 2 |
| RNASEL | G1347 | 5 | hsa-miR-136-5p | M034 | 2 |
| ELP2 | G666 | 5 | hsa-miR-143-3p | M036 | 2 |
| NR3C1 | G1169 | 5 | hsa-miR-193b-3p | M051 | 2 |
| SLC15A4 | G1435 | 5 | hsa-miR-33a-5p | M074 | 2 |
| AP3B1 | G885 | 5 | hsa-miR-33b-5p | M075 | 2 |
| CTCF | G1302 | 5 | hsa-miR-505-3p | M084 | 2 |
| ANO6 | G865 | 5 | hsa-miR-630 | M004 | 1 |
| CREB1 | G1156 | 5 | hsa-miR-1260a | M006 | 1 |
| STAT1 | G740 | 5 | hsa-miR-100-5p | M024 | 1 |
| IL6ST | G1258 | 5 | hsa-miR-149-5p | M040 | 1 |
| GAB1 | G1497 | 5 | hsa-miR-210-3p | M058 | 1 |
| AHR | G1422 | 5 | hsa-miR-708-5p | M087 | 1 |
| GJA1 | G1031 | 5 | hsa-miR-93-5p | M089 | 1 |
| PELI1 | G1427 | 5 |  |  |  |
| PPARG | G1039 | 5 |  |  |  |
| MFN1 | G1008 | 5 |  |  |  |
| STAT3 | G1246 | 5 |  |  |  |
| HMGB2 | G1300 | 5 |  |  |  |
| SMARCA2 | G1237 | 5 |  |  |  |
| GSK3B | G1467 | 5 |  |  |  |
| ITGB3 | G1454 | 5 |  |  |  |
| ATF2 | G1226 | 5 |  |  |  |
| DUSP10 | G1489 | 5 |  |  |  |
| RNF135 | G998 | 5 |  |  |  |
| GLRX | G1461 | 5 |  |  |  |
| TRIM37 | G1029 | 5 |  |  |  |
| PPARGC1B | G1390 | 5 |  |  |  |
| HSP90B1 | G1265 | 5 |  |  |  |
| JAK2 | G607 | 4 |  |  |  |
| AXL | G684 | 4 |  |  |  |
| BCL10 | G1381 | 4 |  |  |  |
| IL1RAP | G769 | 4 |  |  |  |
| INSIG1 | G1308 | 4 |  |  |  |
| CTNNAL1 | G820 | 4 |  |  |  |
| CYLD | G1408 | 4 |  |  |  |
| YWHAE | G655 | 4 |  |  |  |
| ULK1 | G1346 | 4 |  |  |  |
| SMARCE1 | G821 | 4 |  |  |  |
| MAP3K3 | G1480 | 4 |  |  |  |
| DNM1L | G751 | 4 |  |  |  |
| GOPC | G823 | 4 |  |  |  |
| PPP1CC | G1196 | 4 |  |  |  |
| OTUD7B | G1236 | 4 |  |  |  |
| RCAN1 | G1471 | 4 |  |  |  |
| IRF1 | G975 | 4 |  |  |  |
| IRF8 | G1173 | 4 |  |  |  |
| EDIL3 | G1242 | 4 |  |  |  |
| IL6 | G641 | 4 |  |  |  |
| MYH9 | G1001 | 4 |  |  |  |
| UBQLN1 | G696 | 4 |  |  |  |
| MAP3K4 | G879 | 4 |  |  |  |
| RGMB | G699 | 4 |  |  |  |
| SUGT1 | G757 | 4 |  |  |  |
| PMAIP1 | G653 | 4 |  |  |  |
| CALCOCO2 | G833 | 4 |  |  |  |
| KDM4A | G1294 | 4 |  |  |  |
| ITGAV | G1485 | 4 |  |  |  |
| PELI2 | G807 | 4 |  |  |  |
| CCR7 | G1451 | 4 |  |  |  |
| RANBP9 | G920 | 4 |  |  |  |
| ERAP1 | G987 | 4 |  |  |  |
| ETS1 | G1007 | 4 |  |  |  |
| IRF2 | G1342 | 4 |  |  |  |
| DDX21 | G730 | 4 |  |  |  |
| ABL1 | G1393 | 4 |  |  |  |
| NUP153 | G1399 | 4 |  |  |  |
| YY1 | G1180 | 4 |  |  |  |
| SMAD7 | G1431 | 4 |  |  |  |
| THBS1 | G1229 | 4 |  |  |  |
| ELAVL1 | G1398 | 4 |  |  |  |
| GNAI3 | G1257 | 4 |  |  |  |
| SOCS3 | G1387 | 4 |  |  |  |
| RASGEF1B | G1437 | 4 |  |  |  |
| ATM | G1152 | 4 |  |  |  |
| PRKCD | G1161 | 4 |  |  |  |
| C1QBP | G683 | 3 |  |  |  |
| MAP3K8 | G186 | 3 |  |  |  |
| CDC42 | G541 | 3 |  |  |  |
| YBX1 | G827 | 3 |  |  |  |
| G3BP1 | G676 | 3 |  |  |  |
| PTPN11 | G1050 | 3 |  |  |  |
| PCBP2 | G640 | 3 |  |  |  |
| RNF125 | G496 | 3 |  |  |  |
| ILF3 | G886 | 3 |  |  |  |
| TRIM14 | G252 | 3 |  |  |  |
| TRIM27 | G750 | 3 |  |  |  |
| NR4A3 | G1348 | 3 |  |  |  |
| IRF4 | G416 | 3 |  |  |  |
| PIK3R1 | G760 | 3 |  |  |  |
| IL15 | G814 | 3 |  |  |  |
| MTOR | G918 | 3 |  |  |  |
| GATA3 | G1421 | 3 |  |  |  |
| OAS3 | G797 | 3 |  |  |  |
| CFLAR | G871 | 3 |  |  |  |
| TOMM70A | G572 | 3 |  |  |  |
| RCOR1 | G1197 | 3 |  |  |  |
| TRIM65 | G813 | 3 |  |  |  |
| CASP8 | G845 | 3 |  |  |  |
| SIAH2 | G1441 | 3 |  |  |  |
| NFE2L2 | G1409 | 3 |  |  |  |
| TRIM71 | G430 | 3 |  |  |  |
| CTNNB1 | G479 | 3 |  |  |  |
| ASCC3 | G624 | 3 |  |  |  |
| CORO2A | G692 | 3 |  |  |  |
| HMGB1 | G1470 | 3 |  |  |  |
| CISH | G1305 | 3 |  |  |  |
| SDC4 | G1033 | 3 |  |  |  |
| IRAK1 | G1063 | 3 |  |  |  |
| TRIM24 | G926 | 3 |  |  |  |
| NLRC5 | G772 | 3 |  |  |  |
| MAPK14 | G997 | 3 |  |  |  |
| SF3A1 | G524 | 3 |  |  |  |
| CTSB | G1177 | 3 |  |  |  |
| MID1 | G748 | 3 |  |  |  |
| XRCC5 | G762 | 3 |  |  |  |
| KIAA0226 | G1307 | 3 |  |  |  |
| CCNA2 | G1329 | 3 |  |  |  |
| SEMA3A | G1054 | 3 |  |  |  |
| TRAF6 | G1062 | 3 |  |  |  |
| MAPKAPK2 | G1397 | 3 |  |  |  |
| ELF1 | G764 | 3 |  |  |  |
| FER | G786 | 3 |  |  |  |
| EPS8 | G1472 | 3 |  |  |  |
| THRB | G1052 | 3 |  |  |  |
| PTGS2 | G922 | 3 |  |  |  |
| ARL5B | G875 | 3 |  |  |  |
| MFF | G1487 | 3 |  |  |  |
| MAP3K14 | G974 | 3 |  |  |  |
| REST | G1333 | 3 |  |  |  |
| SIVA1 | G1411 | 3 |  |  |  |
| STAT5B | G1301 | 3 |  |  |  |
| MIF | G063 | 2 |  |  |  |
| MYC | G069 | 2 |  |  |  |
| AZI2 | G861 | 2 |  |  |  |
| BIRC3 | G278 | 2 |  |  |  |
| ZC3HAV1 | G583 | 2 |  |  |  |
| PRKX | G1059 | 2 |  |  |  |
| EIF2AK2 | G564 | 2 |  |  |  |
| ATG7 | G659 | 2 |  |  |  |
| PRKCA | G1182 | 2 |  |  |  |
| HSP90AA1 | G537 | 2 |  |  |  |
| PPIA | G348 | 2 |  |  |  |
| CDKN2A | G309 | 2 |  |  |  |
| RAD23A | G503 | 2 |  |  |  |
| XBP1 | G397 | 2 |  |  |  |
| OTUD5 | G717 | 2 |  |  |  |
| KPNA1 | G704 | 2 |  |  |  |
| EGFR | G726 | 2 |  |  |  |
| HNRNPL | G706 | 2 |  |  |  |
| ADAR | G423 | 2 |  |  |  |
| SMAD6 | G335 | 2 |  |  |  |
| FGF7 | G527 | 2 |  |  |  |
| LRRFIP2 | G664 | 2 |  |  |  |
| NOD1 | G329 | 2 |  |  |  |
| COPS8 | G678 | 2 |  |  |  |
| VASP | G790 | 2 |  |  |  |
| STAT6 | G283 | 2 |  |  |  |
| CD274 | G802 | 2 |  |  |  |
| PURA | G1310 | 2 |  |  |  |
| F2RL1 | G584 | 2 |  |  |  |
| TBKBP1 | G935 | 2 |  |  |  |
| DHCR24 | G996 | 2 |  |  |  |
| CEBPA | G1184 | 2 |  |  |  |
| TAX1BP1 | G766 | 2 |  |  |  |
| TYRO3 | G1249 | 2 |  |  |  |
| TLR4 | G668 | 2 |  |  |  |
| SMAD4 | G680 | 2 |  |  |  |
| OAS2 | G763 | 2 |  |  |  |
| PLA2G4A | G505 | 2 |  |  |  |
| CALM1 | G352 | 2 |  |  |  |
| GNB2 | G1424 | 2 |  |  |  |
| TNFRSF1A | G1337 | 2 |  |  |  |
| HOXA9 | G622 | 2 |  |  |  |
| DDIT3 | G787 | 2 |  |  |  |
| TRIM23 | G626 | 2 |  |  |  |
| IFNGR2 | G570 | 2 |  |  |  |
| NKIRAS2 | G636 | 2 |  |  |  |
| PTK2 | G351 | 2 |  |  |  |
| HSPA1A | G642 | 2 |  |  |  |
| TRIM7 | G545 | 2 |  |  |  |
| TSC22D3 | G412 | 2 |  |  |  |
| CASP6 | G840 | 2 |  |  |  |
| SCARB1 | G1074 | 2 |  |  |  |
| VPS45 | G835 | 2 |  |  |  |
| OTUB2 | G789 | 2 |  |  |  |
| PTCH1 | G500 | 2 |  |  |  |
| SQSTM1 | G652 | 2 |  |  |  |
| RUSC1 | G777 | 2 |  |  |  |
| USP4 | G462 | 2 |  |  |  |
| PLAUR | G862 | 2 |  |  |  |
| LRRFIP1 | G1469 | 2 |  |  |  |
| S100A10 | G643 | 2 |  |  |  |
| AMFR | G517 | 2 |  |  |  |
| DUSP16 | G526 | 2 |  |  |  |
| XIAP | G531 | 2 |  |  |  |
| PRKDC | G718 | 2 |  |  |  |
| TRAF5 | G533 | 2 |  |  |  |
| PPP3CA | G538 | 2 |  |  |  |
| RFTN1 | G779 | 2 |  |  |  |
| PIAS1 | G605 | 2 |  |  |  |
| FADD | G567 | 2 |  |  |  |
| CNOT8 | G1479 | 2 |  |  |  |
| MAPK8 | G1104 | 2 |  |  |  |
| ATF3 | G753 | 2 |  |  |  |
| F2R | G1185 | 2 |  |  |  |
| MERTK | G759 | 2 |  |  |  |
| ZBTB20 | G1304 | 2 |  |  |  |
| PROS1 | G615 | 2 |  |  |  |
| CTLA4 | G749 | 2 |  |  |  |
| SIRPA | G1060 | 2 |  |  |  |
| SOCS1 | G1464 | 2 |  |  |  |
| IRF2BP1 | G1406 | 2 |  |  |  |
| PCBP1 | G1245 | 2 |  |  |  |
| PRKRA | G1332 | 2 |  |  |  |
| CSF2RB | G838 | 2 |  |  |  |
| GRK5 | G847 | 2 |  |  |  |
| HMGB3 | G852 | 2 |  |  |  |
| PDCD1LG2 | G983 | 2 |  |  |  |
| E2F1 | G985 | 2 |  |  |  |
| NKIRAS1 | G989 | 2 |  |  |  |
| RUNX3 | G1037 | 2 |  |  |  |
| C5 | G1044 | 2 |  |  |  |
| ANXA4 | G1084 | 2 |  |  |  |
| SIRT1 | G1335 | 2 |  |  |  |
| STIM1 | G1445 | 2 |  |  |  |
| SLX4 | G1483 | 2 |  |  |  |
| CBL | G1450 | 2 |  |  |  |
| GRN | G009 | 1 |  |  |  |
| AKT1 | G012 | 1 |  |  |  |
| MMP9 | G013 | 1 |  |  |  |
| IFI6 | G020 | 1 |  |  |  |
| EIF4EBP2 | G023 | 1 |  |  |  |
| STAP2 | G028 | 1 |  |  |  |
| PROCR | G033 | 1 |  |  |  |
| IFIT1 | G037 | 1 |  |  |  |
| DDX1 | G044 | 1 |  |  |  |
| USP7 | G045 | 1 |  |  |  |
| NFKBIE | G046 | 1 |  |  |  |
| GNAI2 | G048 | 1 |  |  |  |
| PPP4C | G049 | 1 |  |  |  |
| DOK3 | G055 | 1 |  |  |  |
| CYBA | G058 | 1 |  |  |  |
| POLR2F | G059 | 1 |  |  |  |
| RNF41 | G061 | 1 |  |  |  |
| TUFM | G077 | 1 |  |  |  |
| CD81 | G079 | 1 |  |  |  |
| WDR62 | G080 | 1 |  |  |  |
| TRIM26 | G081 | 1 |  |  |  |
| CTSD | G083 | 1 |  |  |  |
| SLAMF1 | G113 | 1 |  |  |  |
| IRF5 | G115 | 1 |  |  |  |
| AAMP | G120 | 1 |  |  |  |
| FOXO3 | G122 | 1 |  |  |  |
| RPS19 | G129 | 1 |  |  |  |
| MAP3K7 | G130 | 1 |  |  |  |
| IKBKG | G137 | 1 |  |  |  |
| TRIM9 | G151 | 1 |  |  |  |
| RPS6KA5 | G153 | 1 |  |  |  |
| ANXA1 | G155 | 1 |  |  |  |
| NFIL3 | G157 | 1 |  |  |  |
| WNT3A | G165 | 1 |  |  |  |
| CD46 | G174 | 1 |  |  |  |
| BCL2L1 | G181 | 1 |  |  |  |
| NUMBL | G184 | 1 |  |  |  |
| VEGFC | G196 | 1 |  |  |  |
| PIN1 | G198 | 1 |  |  |  |
| PTPRC | G201 | 1 |  |  |  |
| EHMT2 | G206 | 1 |  |  |  |
| FANCC | G222 | 1 |  |  |  |
| DUOX2 | G225 | 1 |  |  |  |
| C1R | G231 | 1 |  |  |  |
| PIK3CG | G237 | 1 |  |  |  |
| HSPA1B | G242 | 1 |  |  |  |
| RORC | G245 | 1 |  |  |  |
| RHEB | G246 | 1 |  |  |  |
| ANXA2 | G253 | 1 |  |  |  |
| TP73 | G255 | 1 |  |  |  |
| TIRAP | G258 | 1 |  |  |  |
| KCNJ8 | G279 | 1 |  |  |  |
| TNFRSF13C | G280 | 1 |  |  |  |
| ECSIT | G286 | 1 |  |  |  |
| PSMB8 | G300 | 1 |  |  |  |
| NEU1 | G304 | 1 |  |  |  |
| PPP1CA | G312 | 1 |  |  |  |
| MASP1 | G313 | 1 |  |  |  |
| MAP2K7 | G317 | 1 |  |  |  |
| MYO18A | G324 | 1 |  |  |  |
| TP53 | G325 | 1 |  |  |  |
| TRIM32 | G326 | 1 |  |  |  |
| PTPN2 | G332 | 1 |  |  |  |
| ACHE | G338 | 1 |  |  |  |
| ANPEP | G339 | 1 |  |  |  |
| REL | G341 | 1 |  |  |  |
| EIF4EBP1 | G345 | 1 |  |  |  |
| CXCL2 | G357 | 1 |  |  |  |
| TRAFD1 | G358 | 1 |  |  |  |
| PIK3C3 | G363 | 1 |  |  |  |
| FLI1 | G365 | 1 |  |  |  |
| CD97 | G372 | 1 |  |  |  |
| TNFRSF12A | G373 | 1 |  |  |  |
| UBE2N | G380 | 1 |  |  |  |
| IRF6 | G396 | 1 |  |  |  |
| LGMN | G405 | 1 |  |  |  |
| AIMP1 | G411 | 1 |  |  |  |
| EIF4E | G414 | 1 |  |  |  |
| LGALS8 | G418 | 1 |  |  |  |
| CNOT4 | G431 | 1 |  |  |  |
| KITLG | G438 | 1 |  |  |  |
| CASP10 | G439 | 1 |  |  |  |
| TPST1 | G440 | 1 |  |  |  |
| CHGA | G442 | 1 |  |  |  |
| NOD2 | G446 | 1 |  |  |  |
| IRAK3 | G447 | 1 |  |  |  |
| CCR6 | G451 | 1 |  |  |  |
| JAM3 | G452 | 1 |  |  |  |
| PIK3CA | G457 | 1 |  |  |  |
| IRAK2 | G460 | 1 |  |  |  |
| TRIM8 | G464 | 1 |  |  |  |
| NUP214 | G466 | 1 |  |  |  |
| HDAC2 | G467 | 1 |  |  |  |
| CYTIP | G473 | 1 |  |  |  |
| IFITM3 | G480 | 1 |  |  |  |
| TRADD | G481 | 1 |  |  |  |
| STUB1 | G483 | 1 |  |  |  |
| IFITM2 | G485 | 1 |  |  |  |
| CASP4 | G486 | 1 |  |  |  |
| GAS6 | G489 | 1 |  |  |  |
| CAV1 | G506 | 1 |  |  |  |
| GATA4 | G509 | 1 |  |  |  |
| TRAF3 | G512 | 1 |  |  |  |
| TNFSF10 | G516 | 1 |  |  |  |
| NFKBIZ | G535 | 1 |  |  |  |
| OPTN | G542 | 1 |  |  |  |
| TRIM22 | G550 | 1 |  |  |  |
| HSPD1 | G558 | 1 |  |  |  |
| COPS5 | G561 | 1 |  |  |  |
| RAG1 | G582 | 1 |  |  |  |
| IL10 | G588 | 1 |  |  |  |
| PLSCR1 | G590 | 1 |  |  |  |
| KHSRP | G610 | 1 |  |  |  |
| C7 | G612 | 1 |  |  |  |
| GPSM1 | G630 | 1 |  |  |  |
| GBP2 | G632 | 1 |  |  |  |
| IKBKB | G637 | 1 |  |  |  |
| HLA-E | G645 | 1 |  |  |  |
| ITCH | G648 | 1 |  |  |  |
| NFKBIB | G654 | 1 |  |  |  |
| CD200R1 | G660 | 1 |  |  |  |
| CR2 | G661 | 1 |  |  |  |
| ELF4 | G669 | 1 |  |  |  |
| DAXX | G671 | 1 |  |  |  |
| CCBP2 | G674 | 1 |  |  |  |
| TRIM66 | G677 | 1 |  |  |  |
| FZD1 | G685 | 1 |  |  |  |
| MALT1 | G686 | 1 |  |  |  |
| ATG12 | G689 | 1 |  |  |  |
| CFH | G698 | 1 |  |  |  |
| PIAS3 | G705 | 1 |  |  |  |
| ZMYND11 | G707 | 1 |  |  |  |
| PIK3AP1 | G708 | 1 |  |  |  |
| PRMT1 | G716 | 1 |  |  |  |
| PARD3 | G720 | 1 |  |  |  |
| KDR | G725 | 1 |  |  |  |
| PLK1 | G727 | 1 |  |  |  |
| GABARAP | G736 | 1 |  |  |  |
| AKAP10 | G746 | 1 |  |  |  |
| PML | G752 | 1 |  |  |  |
| MAPK3 | G754 | 1 |  |  |  |
| RPS6KA4 | G761 | 1 |  |  |  |
| GBP1 | G765 | 1 |  |  |  |
| IL33 | G770 | 1 |  |  |  |
| MX2 | G773 | 1 |  |  |  |
| BIRC5 | G784 | 1 |  |  |  |
| TRIM13 | G793 | 1 |  |  |  |
| TRIM28 | G794 | 1 |  |  |  |
| PTX3 | G795 | 1 |  |  |  |
| LUM | G796 | 1 |  |  |  |
| JUN | G803 | 1 |  |  |  |
| CDK9 | G804 | 1 |  |  |  |
| TLR1 | G806 | 1 |  |  |  |
| TRIM6 | G812 | 1 |  |  |  |
| SREBF2 | G818 | 1 |  |  |  |
| MUL1 | G819 | 1 |  |  |  |
| RIPK2 | G824 | 1 |  |  |  |
| TRIM25 | G826 | 1 |  |  |  |
| FSTL1 | G831 | 1 |  |  |  |
| ZC3H12A | G839 | 1 |  |  |  |
| HMGN2 | G850 | 1 |  |  |  |
| RAB8A | G851 | 1 |  |  |  |
| FOXA2 | G853 | 1 |  |  |  |
| APOBEC3B | G867 | 1 |  |  |  |
| PELI3 | G872 | 1 |  |  |  |
| RXRA | G873 | 1 |  |  |  |
| TRIB3 | G878 | 1 |  |  |  |
| WDFY1 | G881 | 1 |  |  |  |
| HSPA14 | G882 | 1 |  |  |  |
| DUSP1 | G921 | 1 |  |  |  |
| TCEB1 | G924 | 1 |  |  |  |
| ITGA3 | G995 | 1 |  |  |  |
| INPP5D | G1000 | 1 |  |  |  |
| TRIM45 | G1005 | 1 |  |  |  |
| SLAMF7 | G1046 | 1 |  |  |  |
| FSCN1 | G1058 | 1 |  |  |  |
| MTA1 | G1170 | 1 |  |  |  |
| PLCG2 | G1178 | 1 |  |  |  |
| TRIM36 | G1183 | 1 |  |  |  |
| CD53 | G1193 | 1 |  |  |  |
| DHX58 | G1247 | 1 |  |  |  |
| TRIM63 | G1290 | 1 |  |  |  |
| TMEM173 | G1309 | 1 |  |  |  |
| IRAK4 | G1340 | 1 |  |  |  |
| VDR | G1349 | 1 |  |  |  |
| CSK | G1414 | 1 |  |  |  |
| ARRB2 | G1415 | 1 |  |  |  |
| TBK1 | G1420 | 1 |  |  |  |
| CEBPB | G1428 | 1 |  |  |  |
| MAP3K5 | G1442 | 1 |  |  |  |
| CTSK | G1448 | 1 |  |  |  |
| TRIM67 | G1455 | 1 |  |  |  |
| CD86 | G1459 | 1 |  |  |  |
| TNFRSF1B | G1463 | 1 |  |  |  |
| SNCA | G1477 | 1 |  |  |  |
| PLEC | G1478 | 1 |  |  |  |
