# Supplementary material for: Dengue virus causes changes of MicroRNA-genes regulatory network revealing potential targets for antiviral drugs
Source: BMC Syst Biol. 2018 Jan 4;12:2. doi: 10.1186/s12918-017-0518-x (PMC5753465; doi:10.1186/s12918-017-0518-x)
Supplement: Supplementary file 4 — MiRNAs, target gene and function pathway process response to dengue virus infection. (DOCX 56 kb) [file 12918_2017_518_MOESM4_ESM.docx]

**Dengue virus causes Changes of MicroRNA-Genes Regulatory Network revealing potential Targets for Antiviral Drugs.**

**Table S2**

MiRNAs, target gene and function pathway process response to dengue virus infection.

| **Gene symbol** | **ID** | **Degree** | **miRNAs Name** | **ID** | **Degree** | **Function Name** | **ID** | **Degree** |
| --- | --- | --- | --- | --- | --- | --- | --- | --- |
| PTEN | G015 | 21 | hsa-miR-320a | Moo5 | 71 | Protein kinase | F008 | 118 |
| DDX3X | G052 | 19 | hsa-miR-361-5p | Moo12 | 59 | regulation of cell proliferation | F003 | 102 |
| ADRB2 | G053 | 17 | hsa-miR-107 | Moo2 | 58 | regulation of programmed cell death and apoptosis | F001 | 100 |
| VEGFA | G006 | 16 | hsa-miR-142-3p | Moo9 | 45 | response to cytokine stimulus | F004 | 86 |
| PPP3R1 | G117 | 16 | hsa-miR-146b-5p | Moo14 | 43 | protein amino acid phosphorylation | F007 | 86 |
| MAP3K1 | G164 | 15 | hsa-miR-542-3p | Moo16 | 41 | immune response | F002 | 76 |
| IGF1R | G038 | 14 | hsa-miR-140-5p | Moo8 | 38 | protein kinase cascade | F009 | 70 |
| RELA | G045 | 14 | hsa-miR-876-5p | Moo17 | 34 | T cell receptor signaling pathway | F010 | 67 |
| NRAS | G209 | 14 | hsa-miR-375 | Moo3 | 29 | regulation of lymphocyte activation | F006 | 49 |
| CHUK | G266 | 14 | hsa-miR-125a-5p | Moo10 | 28 | Toll-like receptor signaling pathway | F011 | 42 |
| MAPK1 | G016 | 13 | hsa-miR-455-5p | Moo15 | 25 | myeloid cell differentiation | F005 | 38 |
| BCL10 | G027 | 13 | hsa-miR-193a-3p | Moo11 | 24 | RIG-I-like receptor signaling pathway | F012 | 29 |
| ADAM10 | G111 | 13 | hsa-miR-106b-5p | Moo27 | 21 |  |  |  |
| RICTOR | G130 | 13 | hsa-miR-106a-5p | Moo26 | 20 |  |  |  |
| MAPK9 | G177 | 13 | hsa-miR-181a-5p | Moo42 | 12 |  |  |  |
| CCDC88A | G019 | 12 | hsa-miR-19a-3p | Moo52 | 12 |  |  |  |
| PRKCE | G070 | 12 | hsa-miR-19b-3p | Moo53 | 12 |  |  |  |
| SOCS5 | G074 | 12 | hsa-miR-25-3p | Moo65 | 12 |  |  |  |
| IL6 | G082 | 12 | hsa-miR-4500 | Moo80 | 12 |  |  |  |
| IL6ST | G122 | 12 | hsa-miR-92a-3p | Moo86 | 12 |  |  |  |
| S1PR1 | G187 | 12 | hsa-miR-181b-5p | Moo43 | 11 |  |  |  |
| TRAF6 | G201 | 12 | hsa-miR-181c-5p | Moo44 | 11 |  |  |  |
| MAP2K1 | G221 | 12 | hsa-miR-181d-5p | Moo45 | 11 |  |  |  |
| PIK3CB | G278 | 12 | hsa-miR-130a-3p | Moo31 | 10 |  |  |  |
| BCL2 | G010 | 11 | hsa-miR-130b-3p | Moo32 | 10 |  |  |  |
| TMED7 | G013 | 11 | hsa-miR-124-3p | Moo29 | 9 |  |  |  |
| MAP3K7 | G068 | 11 | hsa-miR-15b-5p | Moo41 | 9 |  |  |  |
| RAD21 | G099 | 11 | hsa-miR-30c-5p | Moo69 | 9 |  |  |  |
| CTNNB1 | G102 | 11 | hsa-miR-15a-5p | Moo40 | 8 |  |  |  |
| KAT2B | G108 | 11 | hsa-miR-23c | Moo63 | 8 |  |  |  |
| STAT1 | G113 | 11 | hsa-miR-29a-3p | Moo66 | 8 |  |  |  |
| RB1 | G115 | 11 | hsa-miR-30a-5p | Moo68 | 8 |  |  |  |
| ATG5 | G128 | 11 | hsa-miR-30d-5p | Moo70 | 8 |  |  |  |
| RAC1 | G137 | 11 | hsa-miR-424-5p | Moo77 | 8 |  |  |  |
| RIPK2 | G264 | 11 | hsa-miR-101-3p | Moo24 | 7 |  |  |  |
| NFAT5 | G272 | 11 | hsa-miR-186-5p | Moo47 | 7 |  |  |  |
| STAT5B | G288 | 11 | hsa-miR-23a-3p | Moo61 | 7 |  |  |  |
| TSC1 | G044 | 10 | hsa-miR-23b-3p | Moo62 | 7 |  |  |  |
| TLR4 | G106 | 10 | hsa-miR-302a-3p | Moo67 | 7 |  |  |  |
| IL1A | G109 | 10 | hsa-miR-451a | Moo1 | 6 |  |  |  |
| IRAK1 | G133 | 10 | hsa-miR-324-5p | Moo13 | 6 |  |  |  |
| SOCS6 | G140 | 10 | hsa-let-7f-5p | Moo21 | 6 |  |  |  |
| BCL11B | G225 | 10 | hsa-miR-148b-3p | Moo37 | 6 |  |  |  |
| EDN1 | G269 | 10 | hsa-miR-152-3p | Moo39 | 6 |  |  |  |
| PRKCD | G284 | 10 | hsa-let-7b-5p | Moo18 | 5 |  |  |  |
| CDK6 | G003 | 9 | hsa-let-7c-5p | Moo19 | 5 |  |  |  |
| HIF1A | G007 | 9 | hsa-let-7d-5p | Moo20 | 5 |  |  |  |
| AKT1 | G011 | 9 | hsa-let-7i-5p | Moo22 | 5 |  |  |  |
| KLF4 | G014 | 9 | hsa-miR-183-5p | Moo46 | 5 |  |  |  |
| PRKCA | G029 | 9 | hsa-miR-24-3p | Moo64 | 5 |  |  |  |
| CDKN2A | G036 | 9 | hsa-miR-365a-3p | Moo75 | 5 |  |  |  |
| PIK3R1 | G058 | 9 | hsa-miR-7-5p | Moo84 | 5 |  |  |  |
| TNFAIP3 | G076 | 9 | hsa-miR-1260b | Moo7 | 4 |  |  |  |
| PTPRC | G105 | 9 | hsa-miR-145-5p | Moo35 | 4 |  |  |  |
| SIAH1 | G107 | 9 | hsa-miR-196a-5p | Moo50 | 4 |  |  |  |
| GAB1 | G131 | 9 | hsa-miR-223-3p | Moo60 | 4 |  |  |  |
| MAPK8 | G206 | 9 | hsa-miR-340-5p | Moo74 | 4 |  |  |  |
| THBS1 | G226 | 9 | hsa-miR-374a-5p | Moo76 | 4 |  |  |  |
| JAK1 | G247 | 9 | hsa-miR-4306 | Moo79 | 4 |  |  |  |
| SOCS3 | G249 | 9 | hsa-miR-138-5p | Moo34 | 3 |  |  |  |
| MAP3K12 | G271 | 9 | hsa-miR-146a-5p | Moo36 | 3 |  |  |  |
| JAK2 | G004 | 8 | hsa-miR-31-5p | Moo71 | 3 |  |  |  |
| CRKL | G008 | 8 | hsa-miR-425-5p | Moo78 | 3 |  |  |  |
| ITGB1 | G025 | 8 | hsa-miR-4644 | Moo81 | 3 |  |  |  |
| EGFR | G055 | 8 | hsa-miR-935 | Moo6 | 2 |  |  |  |
| IL15 | G066 | 8 | hsa-miR-136-5p | Moo33 | 2 |  |  |  |
| TRIB2 | G069 | 8 | hsa-miR-190a-5p | Moo48 | 2 |  |  |  |
| BECN1 | G075 | 8 | hsa-miR-205-5p | Moo54 | 2 |  |  |  |
| CASP8 | G088 | 8 | hsa-miR-21-5p | Moo55 | 2 |  |  |  |
| GJA1 | G142 | 8 | hsa-miR-22-3p | Moo57 | 2 |  |  |  |
| PPARG | G143 | 8 | hsa-miR-33a-5p | Moo72 | 2 |  |  |  |
| STAT3 | G153 | 8 | hsa-miR-33b-5p | Moo73 | 2 |  |  |  |
| CASP7 | G172 | 8 | hsa-miR-630 | Moo4 | 1 |  |  |  |
| GSK3B | G178 | 8 | hsa-miR-100-5p | Moo23 | 1 |  |  |  |
| PRKDC | G180 | 8 | hsa-miR-103a-3p | Moo25 | 1 |  |  |  |
| NOD2 | G185 | 8 | hsa-miR-10a-5p | Moo28 | 1 |  |  |  |
| PIK3CA | G189 | 8 | hsa-miR-125b-5p | Moo30 | 1 |  |  |  |
| IL10 | G223 | 8 | hsa-miR-149-5p | Moo38 | 1 |  |  |  |
| MALT1 | G239 | 8 | hsa-miR-193b-3p | Moo49 | 1 |  |  |  |
| JUN | G260 | 8 | hsa-miR-197-3p | Moo51 | 1 |  |  |  |
| MAP3K8 | G017 | 7 | hsa-miR-210-3p | Moo56 | 1 |  |  |  |
| MAP2K4 | G020 | 7 | hsa-miR-221-3p | Moo58 | 1 |  |  |  |
| AXL | G023 | 7 | hsa-miR-222-3p | Moo59 | 1 |  |  |  |
| MFN2 | G048 | 7 | hsa-miR-505-3p | Moo82 | 1 |  |  |  |
| RNASEL | G060 | 7 | hsa-miR-513a-5p | Moo83 | 1 |  |  |  |
| IRF1 | G064 | 7 | hsa-miR-708-5p | Moo85 | 1 |  |  |  |
| MTOR | G067 | 7 | hsa-miR-93-5p | Moo87 | 1 |  |  |  |
| IKBKG | G073 | 7 |  |  |  |  |  |  |
| MAP3K4 | G086 | 7 |  |  |  |  |  |  |
| AP3B1 | G089 | 7 |  |  |  |  |  |  |
| VLDLR | G095 | 7 |  |  |  |  |  |  |
| ABCA1 | G104 | 7 |  |  |  |  |  |  |
| CREB1 | G112 | 7 |  |  |  |  |  |  |
| PIK3CG | G119 | 7 |  |  |  |  |  |  |
| LGR4 | G136 | 7 |  |  |  |  |  |  |
| MAPK14 | G150 | 7 |  |  |  |  |  |  |
| TP53 | G155 | 7 |  |  |  |  |  |  |
| IRAK2 | G191 | 7 |  |  |  |  |  |  |
| SAMHD1 | G204 | 7 |  |  |  |  |  |  |
| ABL1 | G207 | 7 |  |  |  |  |  |  |
| F2R | G212 | 7 |  |  |  |  |  |  |
| C5 | G279 | 7 |  |  |  |  |  |  |
| NFATC3 | G282 | 7 |  |  |  |  |  |  |
| ATM | G283 | 7 |  |  |  |  |  |  |
| TBK1 | G293 | 7 |  |  |  |  |  |  |
| CD86 | G297 | 7 |  |  |  |  |  |  |
| MIF | G001 | 6 |  |  |  |  |  |  |
| MYC | G002 | 6 |  |  |  |  |  |  |
| GATA6 | G018 | 6 |  |  |  |  |  |  |
| CDC42 | G026 | 6 |  |  |  |  |  |  |
| NUMB | G030 | 6 |  |  |  |  |  |  |
| IL1RAP | G031 | 6 |  |  |  |  |  |  |
| MAP3K3 | G051 | 6 |  |  |  |  |  |  |
| IRF4 | G057 | 6 |  |  |  |  |  |  |
| NOD1 | G071 | 6 |  |  |  |  |  |  |
| NR3C1 | G072 | 6 |  |  |  |  |  |  |
| MYH9 | G084 | 6 |  |  |  |  |  |  |
| CEBPA | G097 | 6 |  |  |  |  |  |  |
| SMAD4 | G110 | 6 |  |  |  |  |  |  |
| CALCOCO2 | G123 | 6 |  |  |  |  |  |  |
| AHR | G132 | 6 |  |  |  |  |  |  |
| TNFRSF13C | G135 | 6 |  |  |  |  |  |  |
| MAP2K7 | G151 | 6 |  |  |  |  |  |  |
| CCR7 | G159 | 6 |  |  |  |  |  |  |
| SQSTM1 | G162 | 6 |  |  |  |  |  |  |
| ETS1 | G170 | 6 |  |  |  |  |  |  |
| IRAK3 | G186 | 6 |  |  |  |  |  |  |
| ATF2 | G192 | 6 |  |  |  |  |  |  |
| FADD | G196 | 6 |  |  |  |  |  |  |
| TRADD | G198 | 6 |  |  |  |  |  |  |
| CAV1 | G208 | 6 |  |  |  |  |  |  |
| HSPD1 | G218 | 6 |  |  |  |  |  |  |
| DUSP10 | G219 | 6 |  |  |  |  |  |  |
| CTLA4 | G228 | 6 |  |  |  |  |  |  |
| SOCS1 | G229 | 6 |  |  |  |  |  |  |
| IKBKB | G231 | 6 |  |  |  |  |  |  |
| PTGS2 | G241 | 6 |  |  |  |  |  |  |
| MAPK3 | G252 | 6 |  |  |  |  |  |  |
| MAP3K14 | G256 | 6 |  |  |  |  |  |  |
| PDCD1LG2 | G273 | 6 |  |  |  |  |  |  |
| INPP5D | G276 | 6 |  |  |  |  |  |  |
| EIF2AK2 | G028 | 5 |  |  |  |  |  |  |
| INSIG1 | G032 | 5 |  |  |  |  |  |  |
| CYLD | G037 | 5 |  |  |  |  |  |  |
| PTPN11 | G039 | 5 |  |  |  |  |  |  |
| TRIM27 | G046 | 5 |  |  |  |  |  |  |
| PPP1CC | G054 | 5 |  |  |  |  |  |  |
| OTUD7B | G056 | 5 |  |  |  |  |  |  |
| STAT6 | G077 | 5 |  |  |  |  |  |  |
| CD274 | G078 | 5 |  |  |  |  |  |  |
| CFLAR | G080 | 5 |  |  |  |  |  |  |
| UBQLN1 | G085 | 5 |  |  |  |  |  |  |
| BCL2L1 | G094 | 5 |  |  |  |  |  |  |
| PMAIP1 | G098 | 5 |  |  |  |  |  |  |
| TNFRSF1A | G124 | 5 |  |  |  |  |  |  |
| TP73 | G125 | 5 |  |  |  |  |  |  |
| CISH | G126 | 5 |  |  |  |  |  |  |
| SDC4 | G129 | 5 |  |  |  |  |  |  |
| ERAP1 | G167 | 5 |  |  |  |  |  |  |
| XRCC5 | G175 | 5 |  |  |  |  |  |  |
| KITLG | G182 | 5 |  |  |  |  |  |  |
| ELF1 | G216 | 5 |  |  |  |  |  |  |
| FER | G220 | 5 |  |  |  |  |  |  |
| RAG1 | G222 | 5 |  |  |  |  |  |  |
| SMAD7 | G224 | 5 |  |  |  |  |  |  |
| DAXX | G238 | 5 |  |  |  |  |  |  |
| PML | G251 | 5 |  |  |  |  |  |  |
| TLR1 | G262 | 5 |  |  |  |  |  |  |
| MUL1 | G263 | 5 |  |  |  |  |  |  |
| E2F1 | G274 | 5 |  |  |  |  |  |  |
| SIVA1 | G286 | 5 |  |  |  |  |  |  |
| MAP3K5 | G296 | 5 |  |  |  |  |  |  |
| SNCA | G299 | 5 |  |  |  |  |  |  |
| C1QBP | G005 | 4 |  |  |  |  |  |  |
| PRKX | G024 | 4 |  |  |  |  |  |  |
| GNAI2 | G034 | 4 |  |  |  |  |  |  |
| RNF125 | G042 | 4 |  |  |  |  |  |  |
| YWHAE | G043 | 4 |  |  |  |  |  |  |
| CD81 | G049 | 4 |  |  |  |  |  |  |
| TBKBP1 | G090 | 4 |  |  |  |  |  |  |
| SIAH2 | G091 | 4 |  |  |  |  |  |  |
| DHCR24 | G093 | 4 |  |  |  |  |  |  |
| TYRO3 | G100 | 4 |  |  |  |  |  |  |
| VEGFC | G101 | 4 |  |  |  |  |  |  |
| HMGB1 | G120 | 4 |  |  |  |  |  |  |
| TIRAP | G127 | 4 |  |  |  |  |  |  |
| TRIM24 | G141 | 4 |  |  |  |  |  |  |
| CASP6 | G149 | 4 |  |  |  |  |  |  |
| UBE2N | G168 | 4 |  |  |  |  |  |  |
| TRAF5 | G181 | 4 |  |  |  |  |  |  |
| CCNA2 | G193 | 4 |  |  |  |  |  |  |
| SEMA3A | G195 | 4 |  |  |  |  |  |  |
| MAPKAPK2 | G202 | 4 |  |  |  |  |  |  |
| TRAF3 | G213 | 4 |  |  |  |  |  |  |
| TNFSF10 | G214 | 4 |  |  |  |  |  |  |
| C7 | G227 | 4 |  |  |  |  |  |  |
| ITCH | G233 | 4 |  |  |  |  |  |  |
| EPS8 | G236 | 4 |  |  |  |  |  |  |
| ELF4 | G237 | 4 |  |  |  |  |  |  |
| PRKRA | G244 | 4 |  |  |  |  |  |  |
| KDR | G248 | 4 |  |  |  |  |  |  |
| HMGB3 | G258 | 4 |  |  |  |  |  |  |
| CDK9 | G261 | 4 |  |  |  |  |  |  |
| RUNX3 | G277 | 4 |  |  |  |  |  |  |
| SLAMF7 | G280 | 4 |  |  |  |  |  |  |
| PLCG2 | G285 | 4 |  |  |  |  |  |  |
| TMEM173 | G289 | 4 |  |  |  |  |  |  |
| IRAK4 | G290 | 4 |  |  |  |  |  |  |
| CSK | G292 | 4 |  |  |  |  |  |  |
| CBL | G295 | 4 |  |  |  |  |  |  |
| GRN | G009 | 3 |  |  |  |  |  |  |
| AZI2 | G021 | 3 |  |  |  |  |  |  |
| BIRC3 | G022 | 3 |  |  |  |  |  |  |
| OTUD5 | G047 | 3 |  |  |  |  |  |  |
| SMAD6 | G059 | 3 |  |  |  |  |  |  |
| FGF7 | G061 | 3 |  |  |  |  |  |  |
| SLAMF1 | G062 | 3 |  |  |  |  |  |  |
| FOXO3 | G065 | 3 |  |  |  |  |  |  |
| PURA | G079 | 3 |  |  |  |  |  |  |
| RPS6KA5 | G081 | 3 |  |  |  |  |  |  |
| CD46 | G092 | 3 |  |  |  |  |  |  |
| PIN1 | G103 | 3 |  |  |  |  |  |  |
| C1R | G116 | 3 |  |  |  |  |  |  |
| PLA2G4A | G118 | 3 |  |  |  |  |  |  |
| DDIT3 | G134 | 3 |  |  |  |  |  |  |
| IFNGR2 | G139 | 3 |  |  |  |  |  |  |
| NKIRAS2 | G144 | 3 |  |  |  |  |  |  |
| PTK2 | G146 | 3 |  |  |  |  |  |  |
| HSPA1A | G147 | 3 |  |  |  |  |  |  |
| SCARB1 | G152 | 3 |  |  |  |  |  |  |
| REL | G157 | 3 |  |  |  |  |  |  |
| PTCH1 | G160 | 3 |  |  |  |  |  |  |
| CXCL2 | G161 | 3 |  |  |  |  |  |  |
| PIK3C3 | G163 | 3 |  |  |  |  |  |  |
| PLAUR | G169 | 3 |  |  |  |  |  |  |
| AIMP1 | G174 | 3 |  |  |  |  |  |  |
| DUSP16 | G176 | 3 |  |  |  |  |  |  |
| XIAP | G179 | 3 |  |  |  |  |  |  |
| CASP10 | G183 | 3 |  |  |  |  |  |  |
| PPP3CA | G184 | 3 |  |  |  |  |  |  |
| CCR6 | G188 | 3 |  |  |  |  |  |  |
| PIAS1 | G190 | 3 |  |  |  |  |  |  |
| CNOT8 | G205 | 3 |  |  |  |  |  |  |
| GATA4 | G210 | 3 |  |  |  |  |  |  |
| ATF3 | G211 | 3 |  |  |  |  |  |  |
| MERTK | G215 | 3 |  |  |  |  |  |  |
| NFKBIB | G234 | 3 |  |  |  |  |  |  |
| CR2 | G235 | 3 |  |  |  |  |  |  |
| CSF2RB | G245 | 3 |  |  |  |  |  |  |
| PARD3 | G246 | 3 |  |  |  |  |  |  |
| GRK5 | G257 | 3 |  |  |  |  |  |  |
| TRIM25 | G265 | 3 |  |  |  |  |  |  |
| RXRA | G267 | 3 |  |  |  |  |  |  |
| TRIB3 | G268 | 3 |  |  |  |  |  |  |
| NKIRAS1 | G275 | 3 |  |  |  |  |  |  |
| DHX58 | G287 | 3 |  |  |  |  |  |  |
| VDR | G291 | 3 |  |  |  |  |  |  |
| CEBPB | G294 | 3 |  |  |  |  |  |  |
| MMP9 | G012 | 2 |  |  |  |  |  |  |
| NFKBIE | G033 | 2 |  |  |  |  |  |  |
| PPP4C | G035 | 2 |  |  |  |  |  |  |
| CYBA | G040 | 2 |  |  |  |  |  |  |
| POLR2F | G041 | 2 |  |  |  |  |  |  |
| CTSD | G050 | 2 |  |  |  |  |  |  |
| IRF5 | G063 | 2 |  |  |  |  |  |  |
| NFIL3 | G083 | 2 |  |  |  |  |  |  |
| WNT3A | G087 | 2 |  |  |  |  |  |  |
| NUMBL | G096 | 2 |  |  |  |  |  |  |
| DUOX2 | G114 | 2 |  |  |  |  |  |  |
| HSPA1B | G121 | 2 |  |  |  |  |  |  |
| ECSIT | G138 | 2 |  |  |  |  |  |  |
| PSMB8 | G145 | 2 |  |  |  |  |  |  |
| MASP1 | G148 | 2 |  |  |  |  |  |  |
| MYO18A | G154 | 2 |  |  |  |  |  |  |
| ACHE | G156 | 2 |  |  |  |  |  |  |
| EIF4EBP1 | G158 | 2 |  |  |  |  |  |  |
| CD97 | G165 | 2 |  |  |  |  |  |  |
| TNFRSF12A | G166 | 2 |  |  |  |  |  |  |
| IRF6 | G171 | 2 |  |  |  |  |  |  |
| LGMN | G173 | 2 |  |  |  |  |  |  |
| HDAC2 | G194 | 2 |  |  |  |  |  |  |
| IFITM3 | G197 | 2 |  |  |  |  |  |  |
| IFITM2 | G199 | 2 |  |  |  |  |  |  |
| CASP4 | G200 | 2 |  |  |  |  |  |  |
| GAS6 | G203 | 2 |  |  |  |  |  |  |
| OPTN | G217 | 2 |  |  |  |  |  |  |
| GBP2 | G230 | 2 |  |  |  |  |  |  |
| HLA-E | G232 | 2 |  |  |  |  |  |  |
| ATG12 | G240 | 2 |  |  |  |  |  |  |
| CFH | G242 | 2 |  |  |  |  |  |  |
| ZMYND11 | G243 | 2 |  |  |  |  |  |  |
| AKAP10 | G250 | 2 |  |  |  |  |  |  |
| RPS6KA4 | G253 | 2 |  |  |  |  |  |  |
| GBP1 | G254 | 2 |  |  |  |  |  |  |
| IL33 | G255 | 2 |  |  |  |  |  |  |
| TRIM13 | G259 | 2 |  |  |  |  |  |  |
| DUSP1 | G270 | 2 |  |  |  |  |  |  |
| FSCN1 | G281 | 2 |  |  |  |  |  |  |
| TNFRSF1B | G298 | 2 |  |  |  |  |  |  |
