# Supplementary material for: Dengue virus causes changes of MicroRNA-genes regulatory network revealing potential targets for antiviral drugs
Source: BMC Syst Biol. 2018 Jan 4;12:2. doi: 10.1186/s12918-017-0518-x (PMC5753465; doi:10.1186/s12918-017-0518-x)
Supplement: Supplementary file 5 — MiRNAs, Immune target gene and function pathway process response to dengue virus treated by RDN with LRD. (DOCX 48 kb) [file 12918_2017_518_MOESM5_ESM.docx]

**Dengue virus causes Changes of MicroRNA-Genes Regulatory Network revealing potential Targets for Antiviral Drugs.**

**Table S3**

MiRNAs, Immune target gene and function pathway process response to dengue virus infection treated by RDN with LRD.

| **Gene symbol** | **ID** | **Degree** | **miRNAs Name** | **ID** | **Degree** | **Function Name** | **ID** | **Degree** |
| --- | --- | --- | --- | --- | --- | --- | --- | --- |
| RELA | Goo204 | 12 | hsa-miR-107 | M001 | 64 | regulation of programmed cell death and apoptosis | F005 | 94 |
| PTEN | Goo8 | 12 | hsa-miR-361-5p | M006 | 58 | Protein kinase | F003 | 87 |
| TRAF6 | Goo158 | 11 | hsa-miR-542-3p | M009 | 48 | regulation of cell proliferation | F001 | 85 |
| MAPK1 | Goo131 | 11 | hsa-miR-146b-5p | M008 | 45 | protein amino acid phosphorylation | F002 | 72 |
| IL6 | Goo56 | 11 | hsa-miR-125a-5p | M005 | 41 | protein kinase cascade | F008 | 64 |
| MAP3K7 | Goo39 | 11 | hsa-miR-93-5p | M038 | 22 | T cell receptor signaling pathway | F009 | 57 |
| MAP3K1 | Goo208 | 10 | hsa-miR-181a-5p | M016 | 14 | immune response | F006 | 57 |
| MALT1 | Goo155 | 10 | hsa-miR-25-3p | M029 | 12 | response to cytokine stimulus | F007 | 55 |
| NRAS | Goo98 | 10 | hsa-miR-130b-3p | M013 | 10 | regulation of lymphocyte activation | F011 | 34 |
| STAT5B | Goo216 | 9 | hsa-miR-324-5p | M007 | 9 | Toll-like receptor signaling pathway | F012 | 32 |
| CHUK | Goo214 | 9 | hsa-miR-29a-3p | M030 | 8 | myeloid cell differentiation | F004 | 31 |
| PIK3CB | Goo188 | 9 | hsa-miR-186-5p | M017 | 8 | RIG-I-like receptor signaling pathway | F010 | 23 |
| IGF1R | Goo159 | 9 | hsa-miR-23a-3p | M027 | 7 |  |  |  |
| TLR4 | Goo149 | 9 | hsa-miR-214-3p | M022 | 7 |  |  |  |
| MAP2K1 | Goo129 | 9 | hsa-miR-141-3p | M015 | 6 |  |  |  |
| RAC1 | Goo123 | 9 | hsa-miR-7-5p | M037 | 5 |  |  |  |
| CTNNB1 | Goo80 | 9 | hsa-miR-24-3p | M028 | 5 |  |  |  |
| PRKCE | Goo41 | 9 | hsa-let-7i-5p | M011 | 5 |  |  |  |
| PIK3CA | Goo224 | 8 | hsa-let-7b-5p | M010 | 5 |  |  |  |
| PRKCA | Goo207 | 8 | hsa-miR-374b-5p | M035 | 4 |  |  |  |
| IL6ST | Goo190 | 8 | hsa-miR-370-3p | M034 | 4 |  |  |  |
| IRAK1 | Goo152 | 8 | hsa-miR-196a-5p | M018 | 4 |  |  |  |
| IL10 | Goo132 | 8 | hsa-miR-1260b | M004 | 4 |  |  |  |
| RB1 | Goo128 | 8 | hsa-miR-31-5p | M031 | 3 |  |  |  |
| HSPD1 | Goo120 | 8 | hsa-miR-335-5p | M033 | 2 |  |  |  |
| MAPK9 | Goo110 | 8 | hsa-miR-22-3p | M024 | 2 |  |  |  |
| MAPK8 | Goo94 | 8 | hsa-miR-216a-5p | M023 | 2 |  |  |  |
| ADAM10 | Goo86 | 8 | hsa-miR-21-5p | M020 | 2 |  |  |  |
| CDKN2A | Goo73 | 8 | hsa-miR-136-5p | M014 | 2 |  |  |  |
| SOCS5 | Goo46 | 8 | hsa-miR-935 | M003 | 2 |  |  |  |
| IKBKG | Goo45 | 8 | hsa-miR-198 | M002 | 2 |  |  |  |
| PIK3R1 | Goo27 | 8 | hsa-miR-513a-5p | M036 | 1 |  |  |  |
| ADRB2 | Goo19 | 8 | hsa-miR-323b-5p | M032 | 1 |  |  |  |
| VEGFA | Goo2 | 8 | hsa-miR-222-3p | M026 | 1 |  |  |  |
| BCL2 | Goo236 | 7 | hsa-miR-221-3p | M025 | 1 |  |  |  |
| PRKCD | Goo200 | 7 | hsa-miR-210-3p | M021 | 1 |  |  |  |
| PPP3R1 | Goo199 | 7 | hsa-miR-197-3p | M019 | 1 |  |  |  |
| NFAT5 | Goo197 | 7 | hsa-miR-125b-5p | M012 | 1 |  |  |  |
| JUN | Goo178 | 7 |  |  |  |  |  |  |
| STAT1 | Goo104 | 7 |  |  |  |  |  |  |
| F2R | Goo102 | 7 |  |  |  |  |  |  |
| TP53 | Goo83 | 7 |  |  |  |  |  |  |
| STAT3 | Goo81 | 7 |  |  |  |  |  |  |
| TNFAIP3 | Goo48 | 7 |  |  |  |  |  |  |
| IL15 | Goo37 | 7 |  |  |  |  |  |  |
| DDX3X | Goo18 | 7 |  |  |  |  |  |  |
| BCL11B | Goo219 | 6 |  |  |  |  |  |  |
| MAPKAPK2 | Goo217 | 6 |  |  |  |  |  |  |
| PDCD1LG2 | Goo209 | 6 |  |  |  |  |  |  |
| IFNAR1 | Goo194 | 6 |  |  |  |  |  |  |
| C5 | Goo189 | 6 |  |  |  |  |  |  |
| EDN1 | Goo184 | 6 |  |  |  |  |  |  |
| MAPK3 | Goo162 | 6 |  |  |  |  |  |  |
| PML | Goo161 | 6 |  |  |  |  |  |  |
| IKBKB | Goo139 | 6 |  |  |  |  |  |  |
| AXL | Goo125 | 6 |  |  |  |  |  |  |
| TSC1 | Goo117 | 6 |  |  |  |  |  |  |
| CAV1 | Goo97 | 6 |  |  |  |  |  |  |
| ABL1 | Goo95 | 6 |  |  |  |  |  |  |
| MAP2K7 | Goo78 | 6 |  |  |  |  |  |  |
| CASP8 | Goo63 | 6 |  |  |  |  |  |  |
| CCDC88A | Goo44 | 6 |  |  |  |  |  |  |
| NOD1 | Goo42 | 6 |  |  |  |  |  |  |
| MTOR | Goo38 | 6 |  |  |  |  |  |  |
| MAP3K8 | Goo26 | 6 |  |  |  |  |  |  |
| EGFR | Goo21 | 6 |  |  |  |  |  |  |
| HIF1A | Goo3 | 6 |  |  |  |  |  |  |
| MAVS | Goo237 | 5 |  |  |  |  |  |  |
| GAB1 | Goo227 | 5 |  |  |  |  |  |  |
| SNCA | Goo226 | 5 |  |  |  |  |  |  |
| TNFRSF1A | Goo222 | 5 |  |  |  |  |  |  |
| PLCG2 | Goo206 | 5 |  |  |  |  |  |  |
| TMED7 | Goo205 | 5 |  |  |  |  |  |  |
| IL1A | Goo203 | 5 |  |  |  |  |  |  |
| CBL | Goo192 | 5 |  |  |  |  |  |  |
| MAP3K12 | Goo185 | 5 |  |  |  |  |  |  |
| RICTOR | Goo183 | 5 |  |  |  |  |  |  |
| MUL1 | Goo182 | 5 |  |  |  |  |  |  |
| TLR1 | Goo180 | 5 |  |  |  |  |  |  |
| HMGB3 | Goo173 | 5 |  |  |  |  |  |  |
| MAP3K14 | Goo171 | 5 |  |  |  |  |  |  |
| MAP2K4 | Goo169 | 5 |  |  |  |  |  |  |
| GSK3B | Goo168 | 5 |  |  |  |  |  |  |
| PTGS2 | Goo157 | 5 |  |  |  |  |  |  |
| SMAD4 | Goo153 | 5 |  |  |  |  |  |  |
| DAXX | Goo151 | 5 |  |  |  |  |  |  |
| SQSTM1 | Goo142 | 5 |  |  |  |  |  |  |
| SOCS1 | Goo135 | 5 |  |  |  |  |  |  |
| CISH | Goo134 | 5 |  |  |  |  |  |  |
| CTLA4 | Goo133 | 5 |  |  |  |  |  |  |
| RAG1 | Goo130 | 5 |  |  |  |  |  |  |
| FER | Goo127 | 5 |  |  |  |  |  |  |
| FADD | Goo124 | 5 |  |  |  |  |  |  |
| PPARG | Goo115 | 5 |  |  |  |  |  |  |
| TRAF3 | Goo103 | 5 |  |  |  |  |  |  |
| CASP7 | Goo99 | 5 |  |  |  |  |  |  |
| GJA1 | Goo89 | 5 |  |  |  |  |  |  |
| TXNIP | Goo85 | 5 |  |  |  |  |  |  |
| GATA6 | Goo84 | 5 |  |  |  |  |  |  |
| MAPK14 | Goo77 | 5 |  |  |  |  |  |  |
| AP3B1 | Goo64 | 5 |  |  |  |  |  |  |
| MAP3K4 | Goo61 | 5 |  |  |  |  |  |  |
| CDC42 | Goo59 | 5 |  |  |  |  |  |  |
| MYH9 | Goo58 | 5 |  |  |  |  |  |  |
| CD274 | Goo51 | 5 |  |  |  |  |  |  |
| TRIB2 | Goo40 | 5 |  |  |  |  |  |  |
| IRF1 | Goo34 | 5 |  |  |  |  |  |  |
| RNASEL | Goo29 | 5 |  |  |  |  |  |  |
| IRF4 | Goo25 | 5 |  |  |  |  |  |  |
| MAP3K3 | Goo17 | 5 |  |  |  |  |  |  |
| CD81 | Goo15 | 5 |  |  |  |  |  |  |
| MFN2 | Goo14 | 5 |  |  |  |  |  |  |
| GNAI2 | Goo11 | 5 |  |  |  |  |  |  |
| KLF4 | Goo7 | 5 |  |  |  |  |  |  |
| CRKL | Goo5 | 5 |  |  |  |  |  |  |
| CDK6 | Goo1 | 5 |  |  |  |  |  |  |
| CSF1R | Goo239 | 4 |  |  |  |  |  |  |
| E2F1 | Goo231 | 4 |  |  |  |  |  |  |
| JAK1 | Goo228 | 4 |  |  |  |  |  |  |
| TMEM173 | Goo218 | 4 |  |  |  |  |  |  |
| ATM | Goo195 | 4 |  |  |  |  |  |  |
| SLAMF7 | Goo191 | 4 |  |  |  |  |  |  |
| ETS1 | Goo181 | 4 |  |  |  |  |  |  |
| CDK9 | Goo179 | 4 |  |  |  |  |  |  |
| ELF4 | Goo150 | 4 |  |  |  |  |  |  |
| EPS8 | Goo148 | 4 |  |  |  |  |  |  |
| XRCC5 | Goo146 | 4 |  |  |  |  |  |  |
| NFKBIB | Goo144 | 4 |  |  |  |  |  |  |
| ITCH | Goo141 | 4 |  |  |  |  |  |  |
| DUSP10 | Goo126 | 4 |  |  |  |  |  |  |
| EIF2AK2 | Goo121 | 4 |  |  |  |  |  |  |
| KAT2B | Goo118 | 4 |  |  |  |  |  |  |
| ATG5 | Goo113 | 4 |  |  |  |  |  |  |
| ELF1 | Goo108 | 4 |  |  |  |  |  |  |
| RAD21 | Goo107 | 4 |  |  |  |  |  |  |
| TNFSF10 | Goo105 | 4 |  |  |  |  |  |  |
| CALCOCO2 | Goo74 | 4 |  |  |  |  |  |  |
| UBQLN1 | Goo60 | 4 |  |  |  |  |  |  |
| STAT6 | Goo50 | 4 |  |  |  |  |  |  |
| OAS3 | Goo49 | 4 |  |  |  |  |  |  |
| NR3C1 | Goo43 | 4 |  |  |  |  |  |  |
| NUMB | Goo36 | 4 |  |  |  |  |  |  |
| SMAD6 | Goo28 | 4 |  |  |  |  |  |  |
| TRIM27 | Goo12 | 4 |  |  |  |  |  |  |
| TNFRSF1B | Goo238 | 3 |  |  |  |  |  |  |
| ADRBK1 | Goo235 | 3 |  |  |  |  |  |  |
| SCAMP5 | Goo234 | 3 |  |  |  |  |  |  |
| SARM1 | Goo233 | 3 |  |  |  |  |  |  |
| CEBPB | Goo225 | 3 |  |  |  |  |  |  |
| PRKRA | Goo221 | 3 |  |  |  |  |  |  |
| SOCS6 | Goo215 | 3 |  |  |  |  |  |  |
| TYRO3 | Goo213 | 3 |  |  |  |  |  |  |
| VDR | Goo212 | 3 |  |  |  |  |  |  |
| DHX58 | Goo210 | 3 |  |  |  |  |  |  |
| CREB1 | Goo198 | 3 |  |  |  |  |  |  |
| RUNX3 | Goo187 | 3 |  |  |  |  |  |  |
| SDC4 | Goo186 | 3 |  |  |  |  |  |  |
| AHR | Goo163 | 3 |  |  |  |  |  |  |
| PMAIP1 | Goo143 | 3 |  |  |  |  |  |  |
| CTSB | Goo138 | 3 |  |  |  |  |  |  |
| ITGB1 | Goo137 | 3 |  |  |  |  |  |  |
| LGR4 | Goo122 | 3 |  |  |  |  |  |  |
| ERAP1 | Goo119 | 3 |  |  |  |  |  |  |
| TRAF5 | Goo112 | 3 |  |  |  |  |  |  |
| DUSP16 | Goo109 | 3 |  |  |  |  |  |  |
| MERTK | Goo106 | 3 |  |  |  |  |  |  |
| ATF3 | Goo101 | 3 |  |  |  |  |  |  |
| GATA4 | Goo100 | 3 |  |  |  |  |  |  |
| CNOT8 | Goo92 | 3 |  |  |  |  |  |  |
| REL | Goo88 | 3 |  |  |  |  |  |  |
| CASP6 | Goo76 | 3 |  |  |  |  |  |  |
| HSPA1A | Goo71 | 3 |  |  |  |  |  |  |
| NKIRAS2 | Goo68 | 3 |  |  |  |  |  |  |
| SIAH2 | Goo67 | 3 |  |  |  |  |  |  |
| RNF125 | Goo66 | 3 |  |  |  |  |  |  |
| TBKBP1 | Goo65 | 3 |  |  |  |  |  |  |
| RPS6KA5 | Goo54 | 3 |  |  |  |  |  |  |
| CFLAR | Goo53 | 3 |  |  |  |  |  |  |
| PURA | Goo52 | 3 |  |  |  |  |  |  |
| BECN1 | Goo47 | 3 |  |  |  |  |  |  |
| FOXO3 | Goo35 | 3 |  |  |  |  |  |  |
| IL1RAP | Goo32 | 3 |  |  |  |  |  |  |
| SLAMF1 | Goo31 | 3 |  |  |  |  |  |  |
| FGF7 | Goo30 | 3 |  |  |  |  |  |  |
| PTPN11 | Goo24 | 3 |  |  |  |  |  |  |
| ARF6 | Goo23 | 3 |  |  |  |  |  |  |
| PPP1CC | Goo20 | 3 |  |  |  |  |  |  |
| INSIG1 | Goo9 | 3 |  |  |  |  |  |  |
| GRN | Goo6 | 3 |  |  |  |  |  |  |
| CCNT1 | Goo4 | 3 |  |  |  |  |  |  |
| NKIRAS1 | Goo232 | 2 |  |  |  |  |  |  |
| ULK1 | Goo230 | 2 |  |  |  |  |  |  |
| VLDLR | Goo229 | 2 |  |  |  |  |  |  |
| SMARCA2 | Goo223 | 2 |  |  |  |  |  |  |
| CCNA2 | Goo220 | 2 |  |  |  |  |  |  |
| CTSS | Goo211 | 2 |  |  |  |  |  |  |
| S1PR1 | Goo202 | 2 |  |  |  |  |  |  |
| HSP90B1 | Goo201 | 2 |  |  |  |  |  |  |
| SAMHD1 | Goo196 | 2 |  |  |  |  |  |  |
| HMGB1 | Goo193 | 2 |  |  |  |  |  |  |
| TRIM28 | Goo177 | 2 |  |  |  |  |  |  |
| TRIM13 | Goo176 | 2 |  |  |  |  |  |  |
| DDIT3 | Goo175 | 2 |  |  |  |  |  |  |
| BIRC5 | Goo174 | 2 |  |  |  |  |  |  |
| GRK5 | Goo172 | 2 |  |  |  |  |  |  |
| IL33 | Goo170 | 2 |  |  |  |  |  |  |
| TAX1BP1 | Goo167 | 2 |  |  |  |  |  |  |
| GBP1 | Goo166 | 2 |  |  |  |  |  |  |
| OAS2 | Goo165 | 2 |  |  |  |  |  |  |
| RPS6KA4 | Goo164 | 2 |  |  |  |  |  |  |
| CFH | Goo160 | 2 |  |  |  |  |  |  |
| ATG12 | Goo156 | 2 |  |  |  |  |  |  |
| C1QBP | Goo154 | 2 |  |  |  |  |  |  |
| CR2 | Goo147 | 2 |  |  |  |  |  |  |
| YWHAE | Goo145 | 2 |  |  |  |  |  |  |
| HLA-E | Goo140 | 2 |  |  |  |  |  |  |
| GBP2 | Goo136 | 2 |  |  |  |  |  |  |
| OPTN | Goo116 | 2 |  |  |  |  |  |  |
| PPP3CA | Goo114 | 2 |  |  |  |  |  |  |
| XIAP | Goo111 | 2 |  |  |  |  |  |  |
| PLA2G4A | Goo96 | 2 |  |  |  |  |  |  |
| PTCH1 | Goo93 | 2 |  |  |  |  |  |  |
| CCR7 | Goo91 | 2 |  |  |  |  |  |  |
| EIF4EBP1 | Goo90 | 2 |  |  |  |  |  |  |
| ACHE | Goo87 | 2 |  |  |  |  |  |  |
| MYO18A | Goo82 | 2 |  |  |  |  |  |  |
| SCARB1 | Goo79 | 2 |  |  |  |  |  |  |
| MASP1 | Goo75 | 2 |  |  |  |  |  |  |
| TSC22D3 | Goo72 | 2 |  |  |  |  |  |  |
| PTK2 | Goo70 | 2 |  |  |  |  |  |  |
| PSMB8 | Goo69 | 2 |  |  |  |  |  |  |
| WNT3A | Goo62 | 2 |  |  |  |  |  |  |
| NFIL3 | Goo57 | 2 |  |  |  |  |  |  |
| ANXA1 | Goo55 | 2 |  |  |  |  |  |  |
| IRF5 | Goo33 | 2 |  |  |  |  |  |  |
| OTUD7B | Goo22 | 2 |  |  |  |  |  |  |
| CTSD | Goo16 | 2 |  |  |  |  |  |  |
| OTUD5 | Goo13 | 2 |  |  |  |  |  |  |
| NFKBIE | Goo10 | 2 |  |  |  |  |  |  |
