# Supplementary material for: Dengue virus causes changes of MicroRNA-genes regulatory network revealing potential targets for antiviral drugs
Source: BMC Syst Biol. 2018 Jan 4;12:2. doi: 10.1186/s12918-017-0518-x (PMC5753465; doi:10.1186/s12918-017-0518-x)
Supplement: Supplementary file 6 — MiRNAs, immune target gene and function pathway process response to dengue virus treated by Acetaminophen. (DOCX 27 kb) [file 12918_2017_518_MOESM6_ESM.docx]

**Dengue virus causes Changes of MicroRNA-Genes Regulatory Network revealing potential Targets for Antiviral Drugs.**

**Table S4**

MiRNAs, immune target gene and function pathway process response to dengue virus treated by Acetaminophen.

| **Gene symbol** | **ID** | **Degree** | **miRNAs Name** | **ID** | **Degree** | **Function Name** | **ID** | **Degree** |
| --- | --- | --- | --- | --- | --- | --- | --- | --- |
| PRKCA | G021 | 8 | hsa-miR-200c-3p | M003 | 10 | regulation of programmed cell death and apoptosis | F007 | 11 |
| MAP3K1 | G014 | 7 | hsa-miR-141-3p | M002 | 6 | regulation of cell proliferation | F003 | 10 |
| JUN | G016 | 7 | hsa-miR-4725-5p | M005 | 5 | protein amino acid phosphorylation | F004 | 10 |
| MAPK14 | G019 | 7 | hsa-miR-935 | M001 | 2 | Protein kinase | F005 | 10 |
| TBK1 | G015 | 6 | hsa-miR-489-3p | M004 | 1 | T cell receptor signaling pathway | F001 | 7 |
| GNAI2 | G002 | 5 |  |  |  | protein kinase cascade | F002 | 7 |
| TSC1 | G004 | 5 |  |  |  | Toll-like receptor signaling pathway | F008 | 4 |
| NRAS | G020 | 5 |  |  |  | myeloid cell differentiation | F006 | 3 |
| CRKL | G011 | 4 |  |  |  | RIG-I-like receptor signaling pathway | F009 | 3 |
| KDR | G018 | 4 |  |  |  | response to cytokine stimulus | F010 | 3 |
| SNCA | G022 | 4 |  |  |  |  |  |  |
| CBL | G003 | 3 |  |  |  |  |  |  |
| TXNIP | G005 | 3 |  |  |  |  |  |  |
| IFNAR1 | G008 | 3 |  |  |  |  |  |  |
| VEGFA | G009 | 3 |  |  |  |  |  |  |
| IP6K1 | G013 | 3 |  |  |  |  |  |  |
| EPS8 | G017 | 3 |  |  |  |  |  |  |
| NFKBIE | G001 | 2 |  |  |  |  |  |  |
| HMGB1 | G006 | 2 |  |  |  |  |  |  |
| TNFAIP3 | G007 | 2 |  |  |  |  |  |  |
| DUSP1 | G010 | 2 |  |  |  |  |  |  |
| NR3C1 | G012 | 2 |  |  |  |  |  |  |
| ZMYND11 | G023 | 2 |  |  |  |  |  |  |
