# Supplementary material for: Dengue virus causes changes of MicroRNA-genes regulatory network revealing potential targets for antiviral drugs
Source: BMC Syst Biol. 2018 Jan 4;12:2. doi: 10.1186/s12918-017-0518-x (PMC5753465; doi:10.1186/s12918-017-0518-x)
Supplement: Supplementary file 7 — MiRNAs, inflammatory target gene and function pathway process response to dengue virus treated by RDN with LRD. (DOCX 29 kb) [file 12918_2017_518_MOESM7_ESM.docx]

**Dengue virus causes Changes of MicroRNA-Genes Regulatory Network revealing potential Targets for Antiviral Drugs.**

**Table S5**

MiRNAs, inflammatory target gene and function pathway process response to dengue virus treated by RDN with LRD.

| **Gene symbol** | **ID** | **Degree** | **miRNAs Name** | **ID** | **Degree** | **Function Name** | **ID** | **Degree** |
| --- | --- | --- | --- | --- | --- | --- | --- | --- |
| IL6 | G009 | 13 | hsa-miR-146b-5p | M006 | 11 | regulation of programmed cell death and apoptosis | F001 | 26 |
| RELA | G034 | 11 | hsa-miR-107 | M001 | 9 | protein kinase cascade | F007 | 21 |
| ADRB2 | G004 | 10 | hsa-miR-361-5p | M004 | 9 | protein amino acid phosphorylation | F005 | 20 |
| VEGFA | G001 | 9 | hsa-miR-542-3p | M007 | 6 | protein kinase | F006 | 20 |
| STAT1 | G016 | 9 | hsa-miR-125a-5p | M003 | 5 | regulation of cell proliferation | F002 | 18 |
| MAPK9 | G017 | 9 | hsa-miR-25-3p | M019 | 3 | Toll-like receptor signaling pathway | F009 | 11 |
| IKBKB | G021 | 9 | hsa-let-7b-5p | M008 | 2 | immune response | F003 | 10 |
| TLR4 | G022 | 9 | hsa-let-7i-5p | M009 | 2 | myeloid cell differentiation | F004 | 10 |
| CHUK | G037 | 9 | hsa-miR-181a-5p | M012 | 2 | T cell receptor signaling pathway | F011 | 9 |
| PRKCE | G007 | 8 | hsa-miR-186-5p | M013 | 2 | inflammatory response | F008 | 8 |
| MAPK14 | G011 | 8 | hsa-miR-29a-3p | M020 | 2 | protein cascade | F012 | 7 |
| EPO | G013 | 8 | hsa-miR-374b-5p | M022 | 2 | RIG-I-like receptor signaling pathway | F013 | 7 |
| MAPK8 | G014 | 8 | hsa-miR-7-5p | M023 | 2 | regulation of lymphocyte | F010 | 2 |
| IL10 | G020 | 8 | hsa-miR-1260b | M002 | 1 |  |  |  |
| KIT | G036 | 8 | hsa-miR-324-5p | M005 | 1 |  |  |  |
| F2R | G015 | 7 | hsa-miR-130b-3p | M010 | 1 |  |  |  |
| FGF2 | G010 | 6 | hsa-miR-141-3p | M011 | 1 |  |  |  |
| MAP2K7 | G012 | 6 | hsa-miR-198 | M014 | 1 |  |  |  |
| PPARG | G018 | 6 | hsa-miR-216a-5p | M015 | 1 |  |  |  |
| ILK | G027 | 6 | hsa-miR-221-3p | M016 | 1 |  |  |  |
| EDN1 | G031 | 6 | hsa-miR-222-3p | M017 | 1 |  |  |  |
| MAPK12 | G035 | 6 | hsa-miR-23a-3p | M018 | 1 |  |  |  |
| APP | G002 | 5 | hsa-miR-31-5p | M021 | 1 |  |  |  |
| BDNF | G003 | 5 |  |  |  |  |  |  |
| IL1A | G033 | 5 |  |  |  |  |  |  |
| TGFA | G039 | 5 |  |  |  |  |  |  |
| DUSP10 | G019 | 4 |  |  |  |  |  |  |
| PTGS2 | G025 | 4 |  |  |  |  |  |  |
| ZFP36 | G026 | 4 |  |  |  |  |  |  |
| TFRC | G005 | 3 |  |  |  |  |  |  |
| TNFRSF11B | G023 | 3 |  |  |  |  |  |  |
| PTGER4 | G024 | 3 |  |  |  |  |  |  |
| AHR | G028 | 3 |  |  |  |  |  |  |
| COL2A1 | G038 | 3 |  |  |  |  |  |  |
| RNF7 | G006 | 2 |  |  |  |  |  |  |
| STS | G008 | 2 |  |  |  |  |  |  |
| KYNU | G029 | 2 |  |  |  |  |  |  |
| BIRC5 | G030 | 2 |  |  |  |  |  |  |
| HMGB1 | G032 | 2 |  |  |  |  |  |  |
