# Supplementary material for: Dengue virus causes changes of MicroRNA-genes regulatory network revealing potential targets for antiviral drugs
Source: BMC Syst Biol. 2018 Jan 4;12:2. doi: 10.1186/s12918-017-0518-x (PMC5753465; doi:10.1186/s12918-017-0518-x)
Supplement: Supplementary file 8 — MiRNAs, inflammatory target gene and function pathway process response to dengue virus treated by Acetaminophen. (DOCX 27 kb) [file 12918_2017_518_MOESM8_ESM.docx]

**Dengue virus causes Changes of MicroRNA-Genes Regulatory Network revealing potential Targets for Antiviral Drugs.**

**Table S6**

MiRNAs, inflammatory target gene and function pathway process response to dengue virus treated by Acetaminophen.

| **Gene symbol** | **ID** | **Degree** | **miRNAS Name** | **ID** | **Degree** | **Function Name** | **ID** | **Degree** |
| --- | --- | --- | --- | --- | --- | --- | --- | --- |
| IL6 | Goo10 | 10 | hsa-miR-107 | M001 | 10 | regulation of programmed cell death and apoptosis | Foo2 | 16 |
| RELA | Goo12 | 10 | hsa-miR-320d | M004 | 6 | protein kinase | Foo6 | 15 |
| ADRB2 | Goo5 | 9 | hsa-let-7i-5p | M005 | 2 | regulation of cell proliferation | Foo1 | 14 |
| MAPK9 | Goo13 | 8 | hsa-miR-181a-5p | M007 | 2 | protein amino acid phosphorylation | Foo5 | 13 |
| EPO | Goo14 | 8 | hsa-miR-181b-5p | M008 | 2 | protein kinase cascade | Foo7 | 12 |
| CHUK | Goo21 | 8 | hsa-miR-186-5p | M009 | 2 | myeloid cell differentiation | Foo3 | 6 |
| F2R | Goo19 | 7 | hsa-miR-302a-3p | M013 | 2 | immune response | Foo4 | 6 |
| KIT | Goo20 | 7 | hsa-miR-374a-5p | M014 | 2 | Toll-like receptor signaling pathway | Foo9 | 5 |
| VEGFA | Goo1 | 6 | hsa-miR-1260b | M002 | 1 | protein cascade | Foo10 | 3 |
| FGF2 | Goo11 | 6 | hsa-miR-324-5p | M003 | 1 | RIG-I-like receptor signaling pathway | Foo11 | 3 |
| IL1A | Goo18 | 6 | hsa-miR-130b-3p | M006 | 1 |  |  |  |
| APP | Goo2 | 5 | hsa-miR-190a-5p | M010 | 1 |  |  |  |
| EDN1 | Goo16 | 5 | hsa-miR-222-3p | M011 | 1 |  |  |  |
| PPARG | Goo17 | 5 | hsa-miR-23a-3p | M012 | 1 |  |  |  |
| IRAK4 | Goo22 | 5 |  |  |  |  |  |  |
| TGFA | Goo24 | 5 |  |  |  |  |  |  |
| PRKCE | Goo8 | 4 |  |  |  |  |  |  |
| TNFRSF11B | Goo15 | 4 |  |  |  |  |  |  |
| BDNF | Goo4 | 3 |  |  |  |  |  |  |
| TFRC | Goo6 | 3 |  |  |  |  |  |  |
| ADK | Goo3 | 2 |  |  |  |  |  |  |
| RNF7 | Goo7 | 2 |  |  |  |  |  |  |
| STS | Goo9 | 2 |  |  |  |  |  |  |
| AHR | Goo23 | 2 |  |  |  |  |  |  |
